# Decorin promotes decidual M1-like macrophage polarization via mitochondrial dysfunction resulting in recurrent pregnancy loss

**DOI:** 10.7150/thno.78467

**Published:** 2022-10-17

**Authors:** Liling Wang, Huan Wang, Jing Luo, Ting Xie, Gil Mor, Aihua Liao

**Affiliations:** 1 Institute of Reproductive Health, Center for Reproductive Medicine, Tongji Medical College, Huazhong University of Science and Technology, Wuhan, P.R. China.; 2 Department of Woman Health Care, Maternal and Child Health Hospital of Hubei Province affiliated in Tongji Medical College, Huazhong University of Science and Technology, Wuhan, P.R. China.; 3 C.S. Mott Center for Human Growth and Development, Wayne State University School of Medicine, Detroit, MI 48201, USA.

**Keywords:** decorin, decidual macrophages, decidual stromal cells, normal pregnancy, recurrent pregnancy loss, mitochondrial metabolism

## Abstract

**Rationale:** Recurrent pregnancy loss (RPL) is a distressing disorder that seriously affects the physical and psychological health of women. RPL is also a sentinel risk marker for future obstetric complications and warrants in-depth investigation. Abnormal polarization and functions of decidual macrophages are associated with RPL; however, the underlying mechanisms remain poorly understood.

**Methods:** Decorin expression, localization, and content in the decidua of women with normal pregnancy (NP) and those with RPL were measured using reverse transcription-quantitative polymerase chain reaction (RT-qPCR), western blotting, immunofluorescence, and enzyme-linked immunosorbent assay. The profiles of decidual macrophage subsets were determined using flow cytometry and immunofluorescence in both groups. The correlation between decorin content and the proportion of decidual macrophage subsets in the decidua of early NP women was determined using Pearson analysis. The effects of decorin on the polarization and functions of macrophages were assessed in an *in-vitro* model of Raw264.7 cells via flow cytometry, western blotting, and RT-qPCR. Moreover, the mitochondrial metabolism in Raw264.7 cells under decorin administration was measured via flow cytometry, western blotting, and immunofluorescence. Thirty-three pregnant mice were included in the *in vivo* model and underwent different treatments. The embryo abortion rate, macrophage phenotype in the spleen and uteri, and placental development were evaluated using flow cytometry and hematoxylin-eosin staining.

**Results:** Decorin, derived from decidual stromal cells, was highly expressed in the decidua of women with RPL. A positive correlation between decorin content and the proportion of M1-like macrophages was also observed in the decidua of early NP women. *In vitro* studies showed that decorin treatment inhibited macrophage polarization to M2-like subsets and boosted the inflammatory response, which was related to enhanced anaerobic glycolysis, increased mitochondrial membrane potential and intracellular reactive oxygen species levels, reduced mitochondrial mass, and activation of the myeloid differentiation primary response 88-nuclear factor-κB signaling pathway. Adoptive transfer of decorin-treated bone marrow-derived macrophages in pregnant C57BL/6 mice increased the embryo absorption, accompanied by impaired fetal vascularization.

**Conclusions:** Decidual stromal cell-derived decorin can polarize decidual macrophages toward the M1 phenotype by regulating mitochondrial metabolism, resulting in the occurrence of RPL.

## Introduction

Recurrent pregnancy loss (RPL) is a serious pregnancy disorder experienced by 1-5% of women trying to conceive, and is defined as two or more consecutive losses before 28 weeks of gestation 1, 2. RPL is a sentinel risk marker for future obstetric complications, including placental abruption, fetal growth restriction, preterm birth, and stillbirth, as well as a predictor for long-term female health problems, including venous thromboembolism and cardiovascular disease 3, 4. Although several risk factors have been associated with RPL, approximately 60% of couples with RPL exhibit no risk factors 1. Therefore, in-depth investigations are needed to understand the etiology of RPL and provide a comprehensive overview of the medical care and management strategies for women with RPL.

Decidual macrophages, the second largest decidual leukocyte population (20-30%), play indispensable roles in regulating implantation 5, trophoblast invasion, angiogenesis, spiral artery remodeling, placentation, and immune responses during pregnancy 6. Similar to other tissue-resident macrophages 7, the phenotype and functions of decidual macrophages are also influenced by the local microenvironment within the maternal-fetal interface. Decidual stromal cells (DSCs), the most abundant cell type at the maternal-fetal interface, induce decidual cyclooxygenase-2^+^ M2-like macrophage differentiation by fructose-1,6-bisphosphate-mediated interleukin (IL)-27 secretion, which promotes decidualization, trophoblast invasion, and maternal-fetal tolerance during early pregnancy 8. An increased proportion of M1-like decidual macrophages is associated with RPL 9. In addition, a pro-senescent decidual response is related to the occurrence of RPL during the peri-implantation window 10. However, the roles of DSCs and their direct induction mechanism in regulating macrophage functions in RPL remain unclear.

Decorin (DCN), a versatile member of the small leucine-rich proteoglycan family, consists of a protein core and glycosaminoglycan (GAG) chain (chondroitin sulfate [CS] or dermatan sulfate [DS]). By directly targeting and antagonizing multiple binding partners, including collagens, thrombospondin-1, multiple receptor tyrosine kinases, growth factors, proteases and other enzymes, and Toll-like receptors (TLRs), DCN is involved in regulating collagen fibrillogenesis, angiostasis, tumor growth, autophagy, and immune responses, making it the “guardian from the matrix” 11. The binding of DCN to collagen is crucial for proper fibril formation and fibrillar spacing 12. By sequestering transforming growth factor (TGF)-β and inhibiting receptor tyrosine kinases, such as epidermal growth factor receptor, Met, and vascular endothelial growth factor receptor 2, DCN inhibits the progression and metastasis of various tumors 13. At the maternal-fetal interface, decidua-derived DCN plays an important role in restraining the proliferation, migration, and invasion of extravillous trophoblasts 14, promoting decidualization 15, and supporting fetal membrane remodeling at the early stage of gestation in a TGF-β-dependent manner 16. Elevated levels of DCN in plasma are potential predictive biomarkers for several pregnancy complications, including preeclampsia and preterm premature rupture of fetal membranes before the onset of clinical signs 14, 17. More importantly, DCN inhibits the proliferation of macrophage colony-stimulating factor (M-CSF)-induced bone marrow-derived macrophages (BMDMs) 18, enhances their survival, impedes the interaction between TGF-β and macrophages 19, and promotes M1-like macrophage polarization by binding to TLR2 and TLR4 20. Recently, exogenous treatment of macrophages with DCN caused marked upregulation of M1-associated genes by TLR4, which is closely associated with the development of Sjogren's syndrome 21. However, whether decidua-derived DCN is associated with RPL and whether the phenotype and functions of decidual macrophages are also modulated by DCN are not yet known.

*In vitro* literature highlights the importance of mitochondrial metabolism in the activity and identity of diverse macrophage populations 22, 23. Lipopolysaccharide (LPS) stimulation boosts the production of interleukin (IL)-6 and tumor necrosis factor (TNF)-α in macrophages, which is dependent on the increase in mitochondrial mass induced by signal transducers and activators of the transcription 2-dynamin 1 like (DNM1L/DRP1) axis 24. In addition, impaired mitochondrial oxidative phosphorylation (OXPHOS) is observed in LPS-activated macrophages, which hampers the plasticity of proinflammatory M1 to anti-inflammatory M2 repolarization 25. In contrast, M2 macrophages utilize glutamine and fatty acid oxidation (FAO) to support their anti-inflammatory and tissue remodeling properties upon IL-4 stimulation. FAO inhibition is sufficient to exacerbate proinflammatory activation and repress the M2 macrophage phenotype 26. The expression levels of several genes associated with cell metabolism and transport are upregulated in decidual macrophages than in peripheral blood monocyte cells 27. Two distinct subsets of decidual macrophages (CD14^+^CD11c^hi^ and CD14^+^CD11c^low^) utilize different metabolic pathways 28, suggesting potential metabolic regulation of the phenotype and functions of macrophages. However, the role of mitochondrial metabolism in decidual macrophage differentiation remains poorly understood.

In the current study, we systematically investigated the relationship between DSC-derived DCN and decidual macrophages at the maternal-fetal interface, and the role of DCN-induced M1-like macrophage polarization via mitochondrial metabolism in the pathogenesis of RPL. We demonstrated that DCN, secreted by DSCs, polarizes macrophages toward an M1-like phenotype. This polarization is related to increasing mitochondrial membrane potential (MMP) and glycolysis, promoting mitochondrial fission mediated by DRP1, enhancing reactive oxygen species (ROS) production, and activating the myeloid differentiation primary response 88 (MyD88)-nuclear factor (NF)-κB signaling pathway. Aberrant high levels of DCN lead to the dysfunction of decidual macrophages in the decidua of women with RPL. The transfer of DCN-treated BMDMs increased mouse embryo absorption during early pregnancy, accompanied by impaired fetal vascularization. Our study demonstrates how factors outside the cells are responsible for regulating the decidual macrophage polarization leading to RPL. These findings can facilitate further research on the regulation of immunometabolism in physiological and pathological pregnancies.

## Materials and Methods

### Clinical sample collection

All procedures involving participants in this study were approved by the Clinical Trial Ethics Committee of Huazhong University of Science and Technology (Wuhan, China; CTEC number: S512 [2018]). All participants were recruited from the Department of Obstetrics and Gynecology at the Maternal and Child Health Hospital of Hubei Province (Wuhan, China) from March, 2019 to December, 2020. Written consent was obtained from each participant prior to participation in the study.

First-trimester human decidual tissues were obtained from 126 women with clinically normal pregnancies (NPs, terminated for non-medical reasons) and 21 women with RPL. All pregnancies were confirmed using blood and ultrasound tests, and women with RPL caused by genetic abnormalities, infections, thyroid dysfunction, anti-phospholipid antibodies, and abnormal uterine anatomy were excluded. Women with RPL who had two or more spontaneous abortions were included in our study.

### Isolation of human decidual macrophages

Leukocytes from decidual tissues were processed, as previously described 28. Briefly, decidual tissues were minced and digested with DNase I (150 µg/mL, D5025; Sigma-Aldrich, Saint Louis, MO, USA), collagenase (1 mg/mL, BS164; Biosharp, China), and hyaluronidase (1 mg/mL, H3506; Sigma-Aldrich, St. Louis, MO, USA) in the Roswell Park Memorial Institute-1640 culture medium. Ten milliliters of this enzyme cocktail was used per 1 g wet weight of tissue, with pulsed digestion of 3 × 20 min at 37 °C with stirring. After each incubation, the tissue was allowed to settle and the supernatant containing the released cells was removed. Finally, the dispersed cells were filtered through a metal sieve and silk, and washed twice with phosphate-buffered saline (PBS, Biosharp, China). The suspensions were loaded onto a Percoll (BS909; Biosharp, China) density gradient to purify the leukocytes. Decidual immune cells with densities of 25-50% were collected.

### Flow cytometry and cell sorting

The cells were stained with fluorophore-conjugated monoclonal antibodies according to established protocols. Briefly, cells were washed and stained with human Trustain Fc X^TM^ (422302; BioLegend, San Diego, CA, USA) or anti-CD16/32 (553142, BD Biosciences, USA) for 10 min at room temperature to block Fc receptors before incubation with fluorescent antibodies for 30 min at 4 °C, followed by two washes. The cells were analyzed using flow cytometry (LSRII; BD Bioscience, USA), and the data were analyzed using the FlowJo software V10 (Tree Star, Ashland, OR, USA). All specific antibodies used are listed in **Table S1.**

Decidual macrophages were sorted via magnetic-activated cell sorting using CD14 microbeads (130-050-201; Miltenyi Biotec, Germany). The cells obtained by positive selection were stained using fluorescein isothiocyanate anti-CD14 (11-0149-42; Thermo Fisher Scientific, USA) and sorted using fluorescence-activated cell sorting. The final purity of CD14^+^ cells from decidual samples was > 80%.

### RNA sequencing data analysis

Freshly sorted CD14^+^ decidual macrophages (> 10^5^ cells) or decidual tissues were lysed using the TRIzol reagent (Life Technologies, Carlsbad, CA, USA) before transport to Novogene on dry ice. In total, 10 decidual macrophage and 20 decidual tissue samples were sequenced. We selected better sequencing samples (seven decidual macrophage samples and all decidual tissue samples) to analyze the differentially expressed genes. Kyoto Encyclopedia of Genes and Genomes and gene set enrichment analyses were performed using R/Bioconductor with the “clusterProfiler” package.

### Western blotting and enzyme-linked immunosorbent assay (ELISA)

Tissue or cell samples were lysed in lysis buffer (Beyotime Biotechnology, Wuhan, China). Samples were separated using sodium dodecyl sulfate-polyacrylamide gel electrophoresis and transferred to polyvinylidene difluoride membranes (Millipore, Bedford, MA, USA), where specific antibodies were used to visualize the proteins. All specific antibodies used are listed in **Table S2.** Relative protein levels were analyzed using the ImageJ software V1.4 (National Institutes of Health, Bethesda, MD, USA). β-Actin was used as a loading control.

Quantification of DCN in the decidua and cell culture supernatants was performed using ELISA (CSB-E16522 h; Cusabio, Wuhan, China), according to the manufacturer's instructions.

### Immunohistochemistry

Tissue blocks from each sample were selected for immunohistochemical analysis. Briefly, the slides were deparaffinized in xylene and rehydrated in a descending alcohol series, followed by washing with distilled water. The sections were subjected to heat-induced antigen retrieval in a citrate buffer for 10 min. Endogenous peroxidase activity in sections was quenched with 3% hydrogen peroxide (H_2_O_2_) in methanol for 10 min. The slides were then blocked with 5% bovine serum albumin in PBS for 1 h at room temperature and incubated with primary antibodies overnight at 4 °C, followed by incubation with the indicated secondary antibodies for 1 h at room temperature. Brightfield images were obtained using an Olympus microscope (TH4-200, Japan), with adjustments for brightness and contrast made using Photoshop CC (Adobe Systems, San Jose, CA, USA). All specific antibodies used are listed in **Table S2.**

### Immunofluorescence staining

Sections were initially treated as described in immunohistochemical analysis. After blocking with 5% bovine serum albumin in PBS for 1 h at room temperature, the slides were incubated with a mixture of the two primary antibodies overnight at 4 °C. After rinsing, the sections were incubated with a mixture of the two secondary antibodies for 2 h at room temperature, and 4, 6-diamino-2-phenylindole was used to stain the cell nuclei. Finally, images were captured using a fluorescence microscope (TH4-200 Olympus, Tokyo, Japan). All specific antibodies used are listed in **Table S2.**

### RNA extraction and reverse transcription-quantitative polymerase chain reaction (RT-qPCR)

Total RNA was extracted using TRIzol reagent, following the manufacturer's instructions. An equal amount of total RNA (1 μg) was treated with gDNA Eraser reagent to eliminate potential genomic DNA and used for cDNA synthesis in a 20-μL reaction system (Yeasen, Shanghai, China). RT-qPCR amplification analysis was performed with 2 μL of cDNA using SYBR Green Master Mix (Yeasen, Shanghai, China) on q225 (Kubo, Beijing, China). All data were normalized to β-actin expression levels. Primer sequences are listed in **Table S3**.

### Immortalized T-HESC culture and treatment

T-HESCs (a gift from the Medical College of Xiamen University, Fujian, China) were cultured in Dulbecco's modified Eagle's medium/F12 medium (Gibco) supplemented with 10% (v/v) charcoal-stripped fetal bovine serum (CS-FBS; Biological Industries, USA), 1% insulin-transferrin-selenium-ethanolamine (51500056; Thermo Fisher Scientific, USA), 500 ng/mL puromycin, 100 U/mL penicillin, and 100 μg/mL streptomycin (Gibco, USA). T-HESCs were grown in an incubator at 37 °C, 5% CO_2_, and constant humidity. For* in vitro* decidualization, T-HESCs were cultured in a medium containing 2% CS-FBS, 10 nM estrogen (E110145; Aladdin, China), 1 μM medroxyprogesterone acetate (M129409; Aladdin, China), and 50 μM cAMP (A122959; Aladdin, China) for six days, as described previously 29. Decidualization was confirmed by observing the cell morphology under a microscope and measuring the expression of prolactin (PRL) and insulin-like growth factor binding protein 1 (IGFBP-1) via RT-qPCR at the indicated time points.

### Mitochondrial mass and morphology assessment

MitoTracker-Green (MTG, C1048; Beyotime Biotechnology) is an MMP-independent dye that can monitor the mitochondria. After treatment, the cells were stained with MTG (100 nM) and analyzed using flow cytometry. Mean fluorescence intensity (MFI) was quantified using the FlowJo software (Tree Star, San Jose, CA, USA).

MitoSpy Orange CMTMRos (MitoSpy, 424803; BioLegend) is a cell-permeant, fluorogenic chemical reagent that is used to label the mitochondria in living cells. After the cells were stained with MitoSpy (50 nM), laser scanning confocal microscopy (LSCM; Olympus, Tokyo, Japan) was used to observe the mitochondrial morphology.

### MMP measurement

JC-1 (C2006; Beyotime Biotechnology) is a lipophilic cationic dye that accumulates in the inner mitochondrial membrane in response to MMP. It can form red fluorescent aggregates in normal mitochondria; however, it transforms into green fluorescent monomers in depolarized mitochondria. The samples were stained with JC-1 for 30 min at 37 °C and analyzed via flow cytometry. Red/green ratio was used as a semiquantitative indicator of MMP.

The samples were also stained with MitoSpy (50 nM) and MTG (100 nM) at 37 °C for 30 min and analyzed using flow cytometry. The ratio of MitoSpy MFI to MTG MFI was assessed.

### Cellular ROS analysis

We used 2,7-dichlorodihydrofluorescein diacetate (DCFH-DA, S0033S; Beyotime Biotechnology, China), an indicator of intracellular oxidants. Briefly, samples were stained with DCFH-DA (10 μM) at 37 °C for 30 min. After washing twice with PBS, the cells were analyzed via flow cytometry and their MFIs were quantified.

### Lentiviral infection

DCN overexpression in 293T cells was induced using a lentiviral system. The human DCN cDNA fragment was cloned into the lenti-sgRNA-mCherry-Puro vector to construct the DCN expression vector. The lenti-sgRNA-mCherry-Puro vector is an mCherry expression vector that served as a control. Both vectors were used to generate the DCN-expressing lentivirus (LV-DCN) and the control mCherry lentivirus (LV-Ctr). The 293T cell lines were infected with LV-DCN (DCN^OE^-293T) or LV-Ctr (Ctr-293T) overnight and selected using puromycin (1.5 μg/mL) for seven days. The supernatants of DCN^OE^-293T, Ctr-293T, and wild-type 293T cells were collected to detect the DCN content.

### Isolation, differentiation, and adoptive transfer of BMDMs

Femur bone marrow from healthy female C57BL/6 mice was extracted and seeded in complete medium supplemented with 20% L929 conditioned media for five days. Fresh supernatant from Ctr-293T cells (Ctr-BMDMs), supernatant from DCN^OE^-293T cells (DCN^OE^-BMDMs), complete medium containing 100 ng/mL LPS (LPS-BMDMs), or fresh complete medium containing 20% L929-conditioned media (M0-BMDMs) was added at day 4 for 24 h. Finally, BMDMs were collected at day 5, and dams on gestational day (GD)6.5 and GD7.5 received the indicated BMDMs suspended in 200 μL PBS by tail vein injection.

### Mice and experimental treatments

All animal procedures were performed in accordance with the approved guidelines of the Institutional Animal Care and Use Committee of Tongji Medical College, Huazhong University of Science and Technology, Wuhan, China ([2019] IACUC Number: 2747). Female C57BL/6 mice were mated with BALB/c males (both purchased from the Animal Center of Tongji Medical College), and the presence of a vaginal plug indicated a GD of 0.5. C57BL/6 dams were randomly divided into five groups: PBS, M0-BMDM, Ctr-BMDM, DCN^OE^-BMDM, and LPS-BMDM groups, and received the indicated BMDMs (2 × 10^6^ in 200 μL PBS) on GD6.5 and GD7.5. Dams in the PBS group were administered 200 μL PBS at the indicated time points. The embryo abortion rate, macrophage phenotype in the spleen and uterus, and placental development were evaluated on GD10.5.

### Statistical analysis

Statistical analyses were performed using GraphPad Prism 8.0 (GraphPad Software Inc., USA). Normality was determined using the Shapiro-Wilk test. For normally distributed data, data are presented as the mean ± standard error of the mean. One-way analysis of variance, followed by the post hoc Tukey's multiple comparisons test, was used to compare three or more groups, while the data between two groups were compared using the Student's *t*-test. Median and interquartile range were used for the data, which were analyzed using the Mann-Whitney *U* test. Statistical significance was set at 0.05. *P < 0.05, **P < 0.01, ***P < 0.0001.

## Results

### DCN abundance is increased in women with RPL

**Figure 1:**
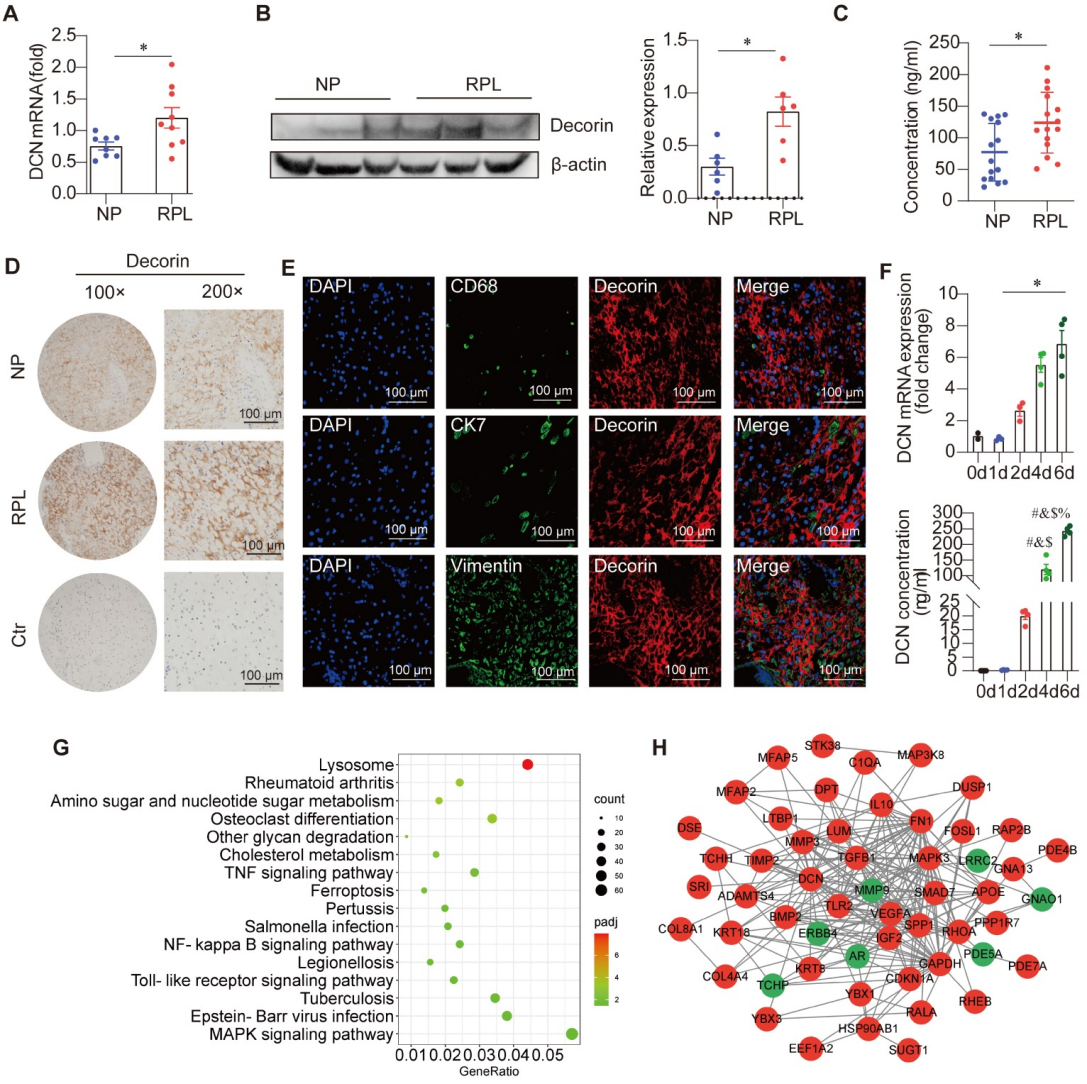
** Decorin (DCN) abundance is increased in patients with recurrent pregnancy loss (RPL). (A-D)** *DCN* mRNA levels, core protein levels, content, and its localization in the decidua of normal pregnancy and RPL groups were determined via reverse transcription-quantitative polymerase chain reaction (RT-qPCR) (n = 8-9), western blotting (n = 6), enzyme-linked immunosorbent assay (ELISA) (n = 15), and immunochemistry, respectively. Scale bar: 100 µm. **(E)** Representative fluorescence images of DCN and CD68, CK7, and Vimentin colocalization in the decidua of women with normal pregnancy (NP). CD68, CK7, and vimentin are markers of total decidual macrophages, trophoblasts, and decidual stromal cells (DSCs), respectively. Nuclei were stained with 4, 6-diamino-2-phenylindole (DAPI). Scale bar: 100 µm. **(F)** DCN mRNA and secreted DCN concentrations in the supernatant of T-HESCs were determined at the indicated time points (0, 1, 2, 4, and 6 d) after decidualization. mRNA expression levels were normalized to β-actin. Statistical analyses were performed via one-way analysis of variance (ANOVA). #, &, $, % indicate the statistically significant differences *vs.* 0, 1, 2, and 4 d, respectively. **(G)** Kyoto Encyclopedia of Genes and Genomes (KEGG) pathway enrichment of differentially expressed genes (DEGs) in the decidua between the NP and RPL groups. **(H)** Protein-protein interaction network of selected DEGs in the decidua. Data represent the mean ± standard error of the mean (SEM) and were analyzed via one-way analysis of variance (ANOVA). *P < 0.05.

To investigate whether aberrant DCN levels were associated with the risk of RPL, we collected decidual samples from women with RPL and those with clinical NP that had been terminated for non-medical reasons. DCN abundance was assessed in the decidua of women with RPL and NP. As shown in **Figure 1A-B**, the *DCN* mRNA and DCN protein core levels were significantly elevated in samples from women with RPL. ELISA data also revealed increased amounts of DCN protein core in women with RPL compared to individuals (123.73 ± 46.35 ng/mL with 77.00 ± 44.14 ng/mL) (**Figure 1C**). In addition, DCN was widely dispersed in the extracellular matrix of the decidua, with deeper staining in women with RPL (**Figure 1D**). These results suggested that DCN may be correlated with the occurrence of RPL.

To confirm the source of DCN in the decidua, we performed immunofluorescence staining of decidual sections from NP groups. We observed few overlapping signals between DCN and CD68 (decidual macrophage marker), CK7 (trophoblast cell marker), and vimentin (DSCs marker) (**Figure 1E**). No DCN-positive staining was observed in trophoblast cells around the villi (**Figure S1A**). However, DCN in the extracellular matrix of the decidua was localized close to vimentin^+^ DSCs (**Figure 1E**). Previous data have shown that DSCs may be the main source of decidual DCN 15. To identify this point, decidualized T-HESC cell lines, primary trophoblast cells, and IL-10/M-CSF-induced peripheral blood mononuclear cell-derived macrophages (PBMC-dM) were used to assess *DCN* mRNA expression levels by RT-qPCR. DCN mRNA levels were significantly higher in decidualised T-HESCs (**Figure S1B**). In addition, a significant time-dependent increase in *DCN* mRNA expression and secreted DCN protein core levels in cell-free supernatants was observed (**Figure 1F**), indicating the main role of DSCs in the secretion of DCN in the decidua. To further validate our findings, we determined the levels of *PRL* and *IGFBP1* mRNA, two genes previously identified as markers of successful decidualization. We found that the mRNA levels of both genes gradually increased in T-HESC cell lines after treatment with decidualizing agents, thus validating our strategy (**Figure S1C**).

Since an excessive inflammatory response is associated with the pathogenesis of RPL 10 and DCN boosts inflammatory activity 20, we also performed RNA sequencing on decidual samples from ten women with RPL and ten NP individuals with NP. Decidua in women with RPL were enriched in various inflammatory pathways, such as the “TNF signaling pathway,” “NF-κB signaling pathway,” “Toll-like receptor signaling pathway,” and “MAPK signaling pathway,” supporting excessive inflammation in RPL (**Figure 1G**). Protein-protein interaction network analysis showed that elevated DCN levels can interact with TLR2 and TGF-β1 (**Figure 1H**). These results suggest that DCN may be part of the inflammatory response and that its abundance further amplifies local inflammation.

### Macrophage polarization toward the M1-like phenotype is associated with RPL

**Figure 2:**
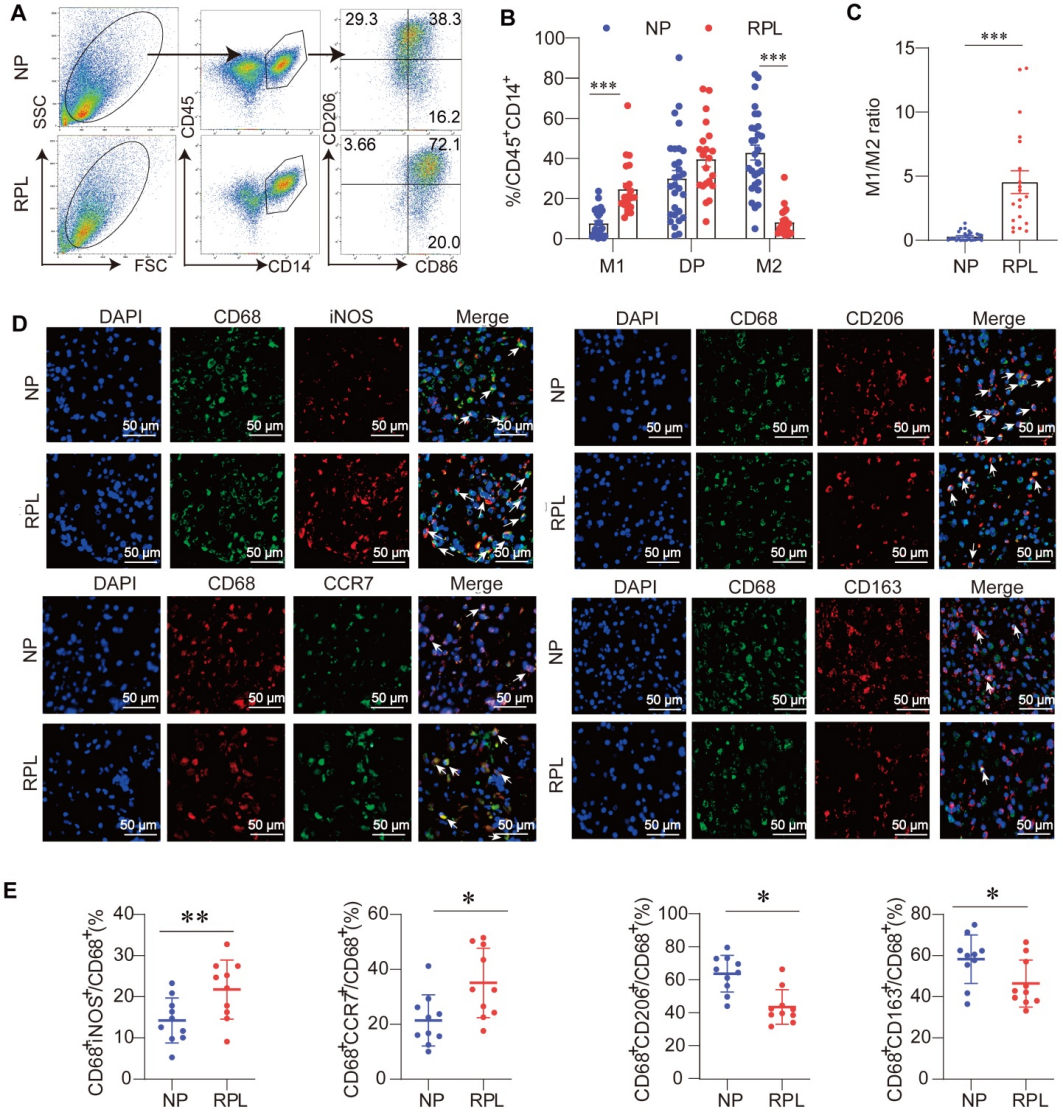
** Decidual macrophages are inclined to become M1-like macrophages in RPL. (A)** Gating strategies and representative flow cytometry analyses of the decidual macrophage subsets in NP and RPL groups. **(B)** Percentages of M1-like, double-positive, and M2-like decidual macrophages were examined in both groups. **(C)** M1-like decidual macrophage/M2-like decidual macrophage ratio in both groups. **(D)** Representative fluorescence images of CD68 and M1-like macrophage markers (inducible nitric oxide synthase [iNOS] and CCR7), and the colocalization of CD68 and M2-like macrophages (CD206 and CD163) in the decidua of both groups. White arrows indicate colocalization in the corresponding fluorescence images. Nuclei were stained with DAPI. Scale bar: 50 µm. **(E)** Quantification of M1-like (CD68^+^iNOS^+^/CD68^+^ and CD68^+^CCR7^+^/CD68^+^) and M2-like (CD68^+^CD206^+^/CD68^+^ and CD68^+^CD163^+^/CD68^+^) cells was performed.

Macrophage polarization is defined as the development of a special phenotype and function suitable for responding to the local microenvironment 7. Traditionally, macrophages are divided into two phenotypes: classically activated (M1) and alternatively activated (M2) macrophages. To assess the proportion of M1-like (CD45^+^CD14^+^CD86^+^CD206^-^) and M2-like (CD45^+^CD14^+^CD86^-^CD206^+^) macrophages in the decidua of women with RPL and NP, decidual immune cells were isolated and subjected to flow cytometry to examine the macrophage phenotypes. The included women were matched for age and BMI (**Table S4**). The results showed that M2-like macrophages dominated the decidua in the NP group (**Figure 2A-B**). Compared with the NP groups, an increased percentage of M1-like macrophages and a decreased percentage of M2-like macrophages were observed in women with RPL, while the proportion of double-positive macrophages (CD45^+^CD14^+^CD86^+^CD206^+^) remained unchanged (**Figure 2A-B**). In addition, a significantly elevated M1/M2 ratio was observed in women with RPL (**Figure 2C**).

To further determine the decidual macrophage profile in both groups, immunofluorescence analysis of CD68 (pan-macrophage marker), inducible nitric oxide synthase (iNOS, M1 marker), chemokine receptor 7 (CCR7, M1 marker), CD206 (M2 marker), and CD163 (M2 marker) in decidual paraffine sections was also performed. As shown in **Figure 2D-E**, we found that the proportions of CD68^+^iNOS^+^ and CD68^+^CCR7^+^ M1-like macrophages in total CD68^+^ macrophages were significantly increased, while CD68^+^CD206^+^ and CD68^+^CD163^+^ M2-like macrophages were lower in women with RPL than in NP individuals. These data support the idea that decidual macrophages exhibit aberrant M1-like macrophage polarization in women with RPL.

### Decidual DCN levels are negatively correlated with the percentage of M2-like macrophages during early pregnancy under physiological conditions

**Figure 3:**
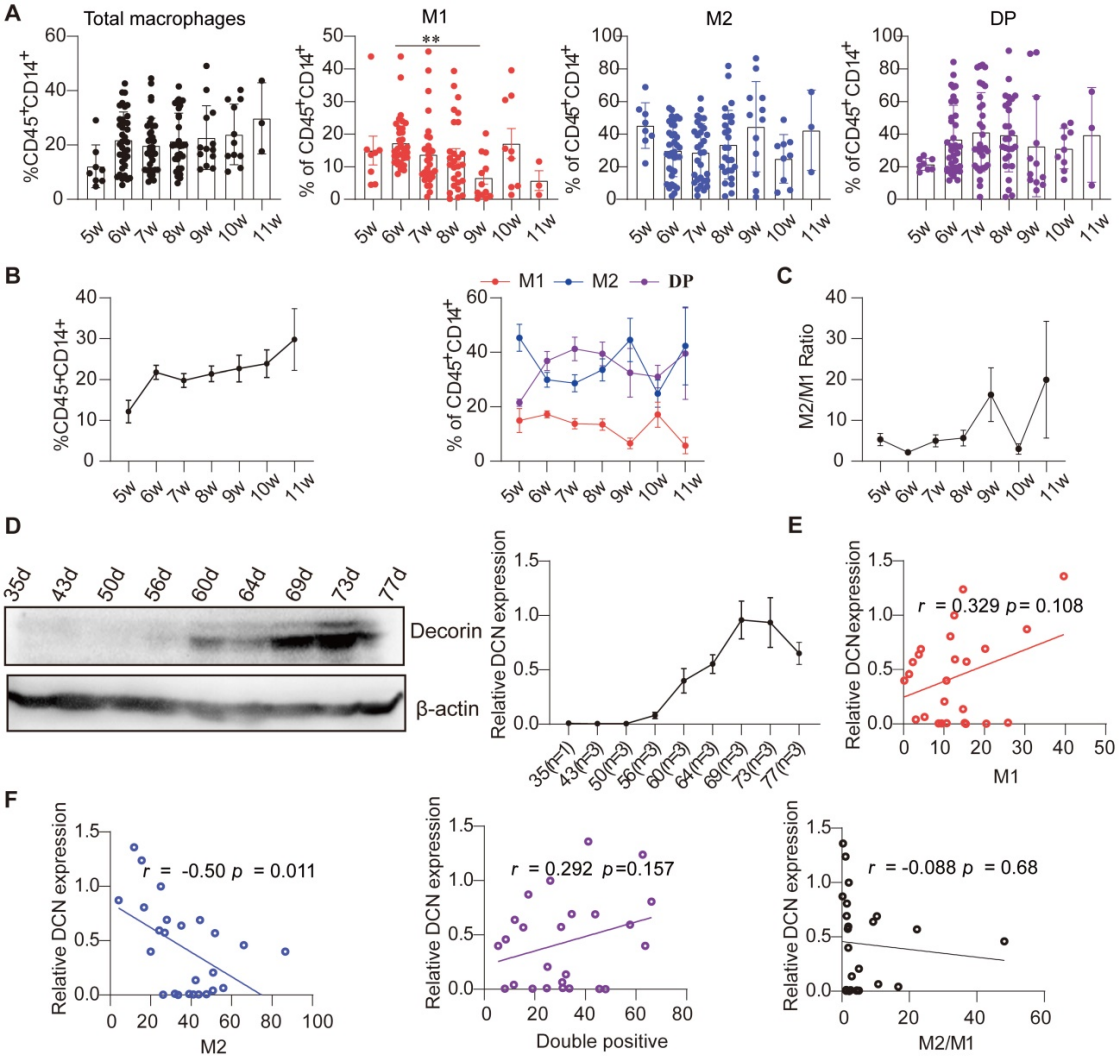
** Dynamic changes in the proportion of decidual macrophage subsets are related to the DCN content in the decidua during early NP. (A and B)** Flow cytometry analysis of the percentages of total, M1-like, double-positive, and M2-like macrophages over the gestational weeks in the decidua of women with early NP. **(C)** M1-like macrophage/M2-like macrophage ratio in the decidua during pregnancy progression. **(D)** Western blotting analysis of DCN core protein in decidua during pregnancy progression. Left panel: Representative western blotting image. The timing of pregnancy termination (day) is shown for each sample, and the number of samples at each pregnancy termination is shown on the horizontal coordinate (right panel). **(E and F)** Pearson correlation analysis of DCN content and percentage of decidual macrophage subsets. (A-C) Data represent the mean ± SEM and were analyzed via one-way ANOVA. *P < 0.05. DP: double-positive macrophages.

Previous studies have shown that DCN is involved in regulating proliferation, apoptosis, adhesion, polarization, and function of macrophages 18, 20. To determine the relationship between the percentage of decidual macrophages and DCN levels, we examined the proportion of decidual macrophage subsets and DCN levels during normal early pregnancy under physiological conditions. A total of 126 decidual samples at different gestational weeks were collected from individuals with NP who had been terminated for non-medical reasons (clinical data are shown in **Table S5**), and the percentage of decidual macrophage subsets was assessed by flow cytometry. The results showed that the proportion of total decidual macrophages (CD45^+^CD14^+^) gradually increased with gestational age (**Figure 3A-B; Table S6**). The proportion of M1-like, M2-like, and double-positive macrophages fluctuated with gestational week, and the M2-like and double-positive macrophage subsets dominated in the decidua during the first trimester (**Figure 3A-B; Table S6**). In addition, the M2/M1 ratio also changed with increasing gestational age, characterized by a slow increase from 6 to 9 weeks, decreasing sharply at 10 weeks, and then returning to the original levels at 11 weeks (**Figure 3C; Table S6**).

We also screened DCN protein core levels during different weeks of pregnancy. Interestingly, DCN protein core expression increased gradually from 8 gestational weeks, peaked at approximately 10 weeks, and then decreased during the first trimester (samples collected from days 35-77) (**Figure 3D**). Pearson analysis showed that the levels of the DCN protein core were positively correlated with the percentage of M1-like and double-positive macrophages but negatively correlated with the percentage of M2-like macrophages and the M2/M1 ratio, especially M2-like macrophages (*r* = -0.50, *P* = 0.011) (**Figure 3E-F**). Taken together, these results indicate that decidual DCN may be involved in the dynamic profile of decidual macrophage subsets during the first trimester, and that high DCN levels during early pregnancy may result in aberrant macrophage polarization in the decidua.

### Exogenous DCN induces M1-like macrophage polarization

**Figure 4:**
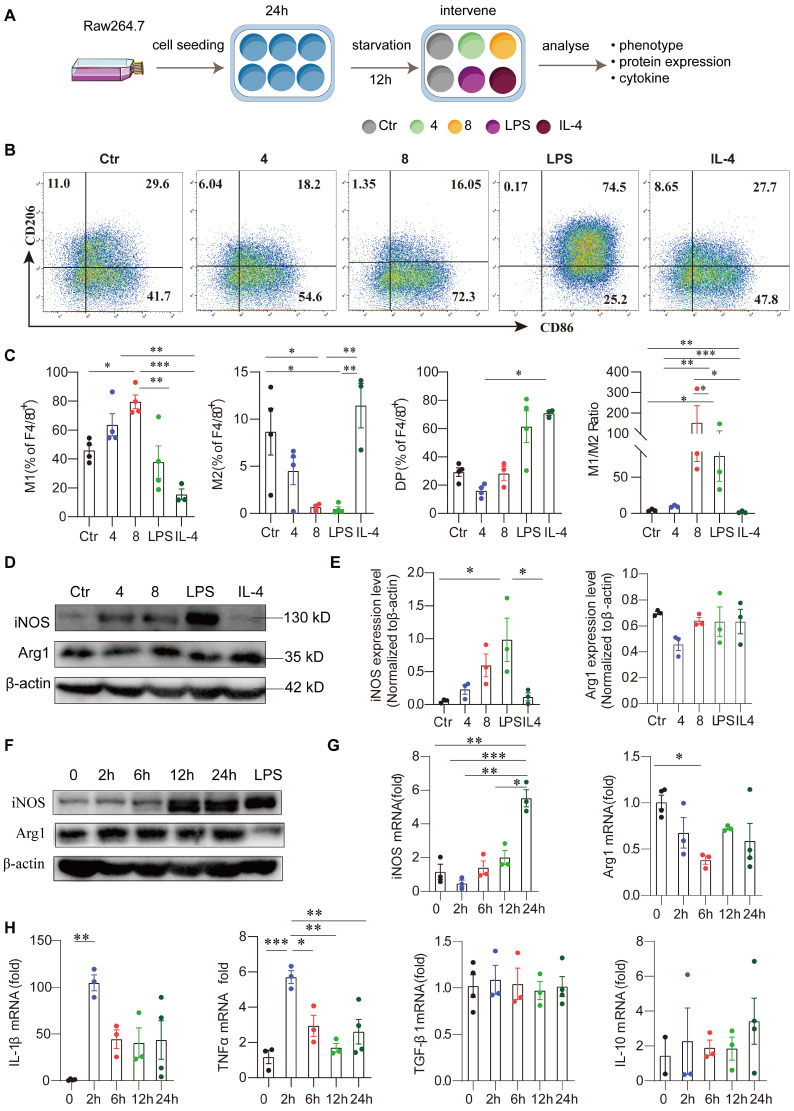
** Exogenous DCN induces M1-like macrophage polarization. (A)** Raw264.7 cells were seeded at 1 × 10^5^ mL/well, cultured for 24 h, and treated with fetal bovine serum (FBS)-free medium for 12 h of starvation in a 12-well round bottom plate. In some wells, different concentrations of DCN (4 and 8 µg/mL), lipopolysaccharide (LPS; 100 ng/mL), and interleukin (IL)-4 (25 ng/mL) were added to the culture system for 24 h. Then, the macrophage phenotype, protein levels of macrophage markers, and mRNA levels of cytokines were detected via flow cytometry, western blotting, and RT-qPCR, respectively. A schematic representation of the cell culture treatment is shown in (A). **(B and C)** Representative images and statistical results of the macrophage subset proportions in different groups. Ctr: without treatment; 4: treated with 4 µg/mL DCN for 24 h; 8: treated with 8 µg/mL DCN for 24 h; LPS and IL-4: treated with 100 ng/mL LPS and 25 ng/mL IL-4 for 24 h. **(D and E)** Representative images and statistical results of iNOS and arginase 1 (ARG1) expression in different groups. **(F)** Representative images of iNOS and ARG1 expression at the indicated time points after DCN (4 µg/mL) treatment. **(G and H)** Statistical analysis of the mRNA levels of macrophage markers (iNOS and *Arg1*), proinflammatory cytokines (*Il-1β* and tumor necrosis factor [*Tnf*]-*α*) and anti-inflammatory cytokines (transforming growth factor [*Tgf*]*-β1* and *Il-10*) at the indicated time points after DCN (4 μg/mL) treatment. Data represent the mean ± SEM and were analyzed via one-way ANOVA. *P < 0.05, **P < 0.01, ***P < 0.001.

To investigate whether DCN directly induces M1-like macrophage polarization, we treated Raw264.7 cells with different concentrations of DCN *in vitro* and determined the phenotypes of macrophages (**Figure 4A**). Flow cytometry analysis showed that 24 h after exogenous DCN treatment, the proportion of M1-like macrophages (F4/80^+^CD86^+^CD206^-^) significantly increased, accounting for approximately 80% of F4/80^+^ macrophages, while the proportion of M2-like macrophages (F4/80^+^CD86^-^CD206^+^) decreased in a dose-dependent manner compared to the control. No obvious changes were observed in the percentage of double-positive macrophages following DCN treatment. DCN treatment also increased the M1/M2 ratio in Raw264.7 cells without statistical significance (**Figure 4B-C**). In addition, DCN increased INOS protein levels but had no significant effect on arginase 1 (ARG1) protein expression (**Figure 4D-E**). Moreover, the effect of DCN on macrophage polarization was different from that in the LPS (M1) and IL-4 (M2) groups but was closer to the phenotypes induced by LPS (**Figure 4B-E**).

Macrophage phenotypic markers (INOS and ARG1) and cytokines were also assessed after stimulation with DCN for varying durations. INOS protein levels significantly increased after 12 h in a time-dependent manner (**Figure 4F**). Although significantly reduced* Arg1* mRNA levels were found at 6 h, no changes in ARG1 protein levels were observed after exogenous DCN treatment (**Figure 4F-G**). DCN induced *Il-1β* mRNA and *TNF-α* mRNA expression in a time-dependent manner compared to the control (0 h). The kinetics of proinflammatory cytokines were similar. They increased rapidly, peaking at 2 h, and then fell rapidly, maintaining high levels within 24 h (**Figure 4H**). However, no significant changes were observed in anti-inflammatory cytokines, such as *Tgf-β1* mRNA and *Il-10* mRNA (**Figure 4H**), suggesting that DCN could induce macrophage polarization to the M1-like proinflammatory phenotype.

### Dysfunctional mitochondrial metabolism determines the inflammatory phenotype of DCN-treated macrophages

**Figure 5:**
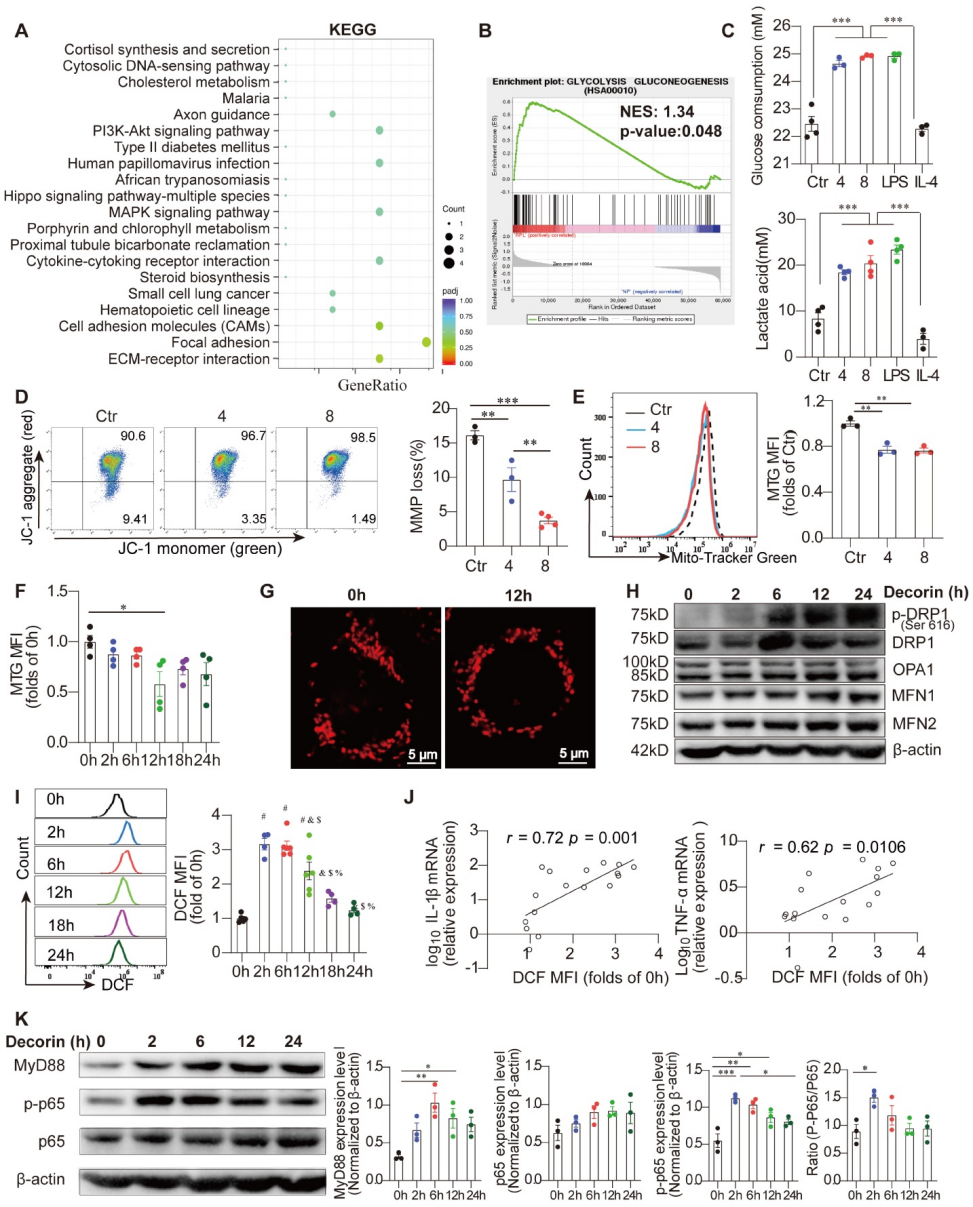
** Dysfunctional mitochondrial metabolism determines the inflammatory phenotype of DCN-treated macrophages. (A and B)** KEGG pathway enrichment analysis and gene set enrichment analysis (GSEA) of DEGs in decidual macrophages between NP and RPL groups. **(C)** Analysis of 24 h glucose consumption and lactate production in the supernatant of differently treated Raw264.7 cells (n = 3-4 for each group). Mitochondrial membrane potential (MMP) was detected using the fluorescent probe, JC-1. JC-1 aggregates, red; JC-1 monomers, green. Representative images and statistical results of MMP are shown in (**D**). **(E)** MitoTracker-Green (MTG)-stained cells were detected via flow cytometry. n = 3. **(F)** MTG mean fluorescence intensity (MFI) was calculated at the indicated time points after DCN (4 µg/mL) treatment. **(G)** Mitospy-stained cells were captured via laser scanning confocal microscopy (LSCM). Scale bars: 5 µm. **(H)** Western blotting of dynamin 1 like (DNM1L/DRP1), p-DRP1, optic atrophy 1 (OPA1), mitofusin (MFN)-1, and MFN2. **(I)** 2,7-dichlorodihydrofluorescein diacetate (DCFH-DA)-stained cells were tested via flow cytometry, and MFIs were calculated at the indicated time points after administration of 4 µg/mL DCN. **(J)** Correlations between DCFH-DA MFI and log_10_*Il-1β* or log_10_*Tnfα* were evaluated via Pearson analysis. Representative protein bands and statistical results of MyD88, p-p65, and p65 are shown in (**K**). Data represent the mean ± SEM and were analyzed via one-way ANOVA. *P < 0.05, **P < 0.01, ***P < 0.001.

Recent studies have indicated that changes in mitochondrial metabolism play an important role in regulating the activation, differentiation, and survival of macrophages in response to various extracellular signals 22. These changes include alterations in oxidative metabolism, MMP, the release of mitochondrial ROS and mitochondrial dynamics 22, 24. Interestingly, transcriptome data indicated that gene sets associated with the glycolysis pathway were enriched in primary decidual macrophages from women with RPL compared to those from women with NP (**Figure 5A**). Primary decidual macrophages in women with RPL were also enriched in focal adhesions and ECM-receptor interactions (**Figure 5B**). More importantly, the culture medium (containing phenol red) of DCN-treated Raw264.7 macrophages seemed to be more acidic than that of control macrophages, similar to LPS-treated macrophages, indicating that their metabolism was altered by DCN. Indeed, both glucose consumption and lactate accumulation were increased in DCN-treated Raw264.7 macrophages, which was similar to the kinetics in the LPS groups (**Figure 5C**). Furthermore, the concentrations of glucose and lactate in the supernatant changed in a time-dependent manner (**Figure S2A**). Taken together, these results suggest that abnormally increased DCN levels promote M1-like macrophage polarization by promoting glycolysis in the decidua of women with RPL.

To investigate whether DCN influences mitochondrial metabolism, we first evaluated the MMP in DCN-treated macrophages using the fluorescent dye JC-1. JC-1 forms red fluorescent aggregates in normal mitochondria and transforms into green fluorescent aggregates in depolarized mitochondria. Flow cytometry results showed that DCN exposure resulted in an abundance of red aggregates of JC-1, which was evidenced by the loss of green fluorescence (**Figure 5D**), demonstrating increased MMP in DCN-treated macrophages. The elevated MitoSpy MFI/MTG MFI ratio also supported increased MMP (**Figure S2B**). In addition, the mitochondrial mass, as indicated by the MTG MFI, showed an obvious decrease in DCN-treated macrophages (**Figure 5E**), with the most pronounced decline at 12 h after DCN intervention (**Figure 5F**). In addition, mitochondrial morphology in DCN-treated macrophages stained with MitoSpy^TM^ orange CMTMRos at 12 h was also assessed by LSCM. Compared to the control macrophages, DCN-treated macrophages contained more mitochondria with a predominantly punctate morphology (**Figure 5G**), indicating mitochondrial fission in DCN-treated macrophages.

To determine whether mitochondrial structural proteins were abnormally expressed in DCN-treated macrophages, we used western blotting and densitometry analyses to quantify DRP1, p-DRP1, optic atrophy 1 (OPA1), mitofusin (MFN)-1, and MFN2 expression. Mitochondrial fission is mainly mediated by DRP1 and mitochondrial fusion is mainly controlled by OPA1 and two mitochondrial outer-membrane-localized proteins, MFN1 and MFN2 23. Here, we observed that DCN significantly enhanced the expression of p-DRP1, but had no effect on OPA1, MFN1, and MFN2 (**Figure 5H**). Meanwhile, ROS, as indicated by DCF MFI, increased 3-fold at 2 h in DCN-treated macrophages, with a time-dependent decline thereafter (**Figure 5I**). Excessive ROS generally increase the production of proinflammatory cytokines in macrophages 30, 31. Pearson's correlation analysis showed that both *Il-1β* mRNA and *Tnf-α* mRNA were positively correlated with ROS levels in DCN-treated macrophages (**Figure 5J**). Therefore, we demonstrated that DCN regulates the phenotype and function of macrophages by reducing mitochondrial MMP and MMP mass, promoting mitochondrial fission, and increasing ROS production.

Given the observation that DCN can interact with TLR2 (**Figure 1G**), we investigated whether DCN could activate the downstream MyD88-NF-κB signaling pathway in Raw264.7 cells. Raw264.7 cells were stimulated with 4 μg/mL DCN for 0, 2, 6, 12, and 24 h. We found that DCN robustly increased the protein expression of MyD88 and p-p65 within 6 h, with a significantly increased p-p65/p65 ratio at 2 h (**Figure 5K**), suggesting that DCN can also activate the MyD88-NF-κB signaling pathway.

### DCN overexpression in 293T cells induces a proinflammatory M1-like phenotype

**Figure 6:**
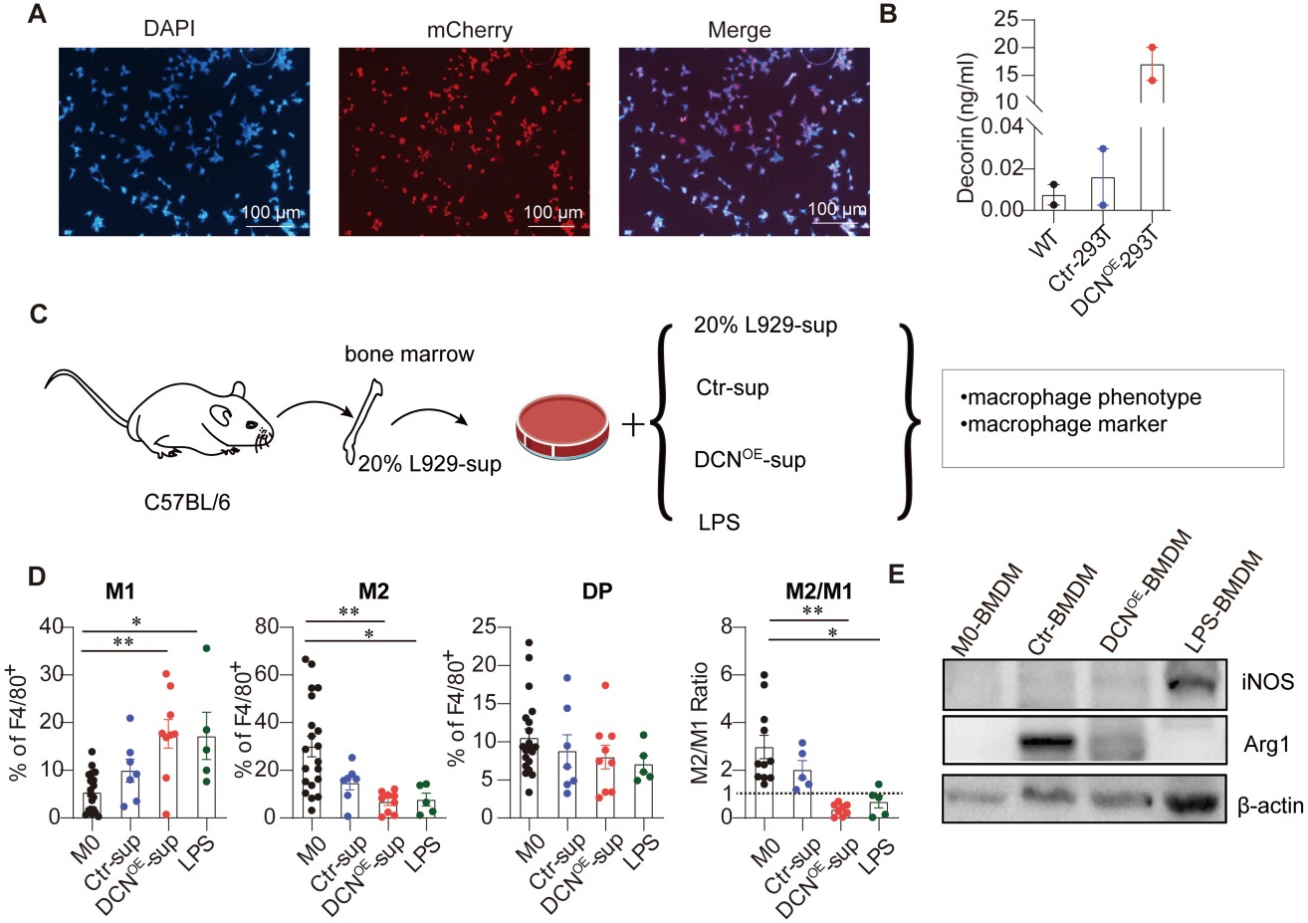
** DCN overexpression in 293T cells induces M1-like polarization. (A)** Representative fluorescence images of mCherry in Ctr-293T cells. **(B)** Concentration of DCN in the supernatant as measured using ELISA. **(C)** Schematic representation of the bone marrow-derived macrophage (BMDM) culture treatment. Fresh supernatant from Ctr-293T cells (Ctr-sup), supernatant from DCN^OE^-293T cells (DCN^OE^-sup), complete medium containing 100 ng/mL LPS, or fresh complete medium containing 20% L929-conditioned media were added at day 4 for 24 h. The macrophage phenotype and expression levels of macrophage markers (iNOS and ARG1) were measured via flow cytometry **(D)** and western blotting **(E)**, respectively.

Given that commercial DCN was not produced, DCN overexpression was established in the 293T (DCN^OE^-293T) cell line. Successful establishment of the DCN^OE^-293T cell line was evidenced by red fluorescence in the nucleus of control lentivirus-transfected 293T (Ctr-293T) cell lines and increased DCN abundance in the supernatant of DCN^OE^-293T cell lines (**Figure 6A-B**). Next, we investigated whether the supernatant of DCN^OE^-293T cells contributed to BMDM polarization to the M1-like phenotype. BMDMs were treated with the supernatant from the Ctr-293T and DCN^OE^-293T cell lines on day 4 for 24 h (**Figure 6C**). We found that the supernatant of DCN^OE^-293T cells significantly increased the percentage of M1-like macrophages and decreased the proportion of M2-like macrophages and the M2/M1 ratio compared with M0-BMDMs (**Figure 6D**). In addition, the supernatant from DCN^OE^-293T cells also reduced ARG1 protein expression in BMDMs compared to that in the supernatant from Ctr-293T cells (**Figure 6E**). Thus, DCN^OE^-293T cell lines were successfully established, and the supernatant could polarize BMDMs into M1-like macrophages.

### Adoptive transfer of DCN-induced macrophages results in embryo absorption and impaired fetal vascularization during early pregnancy

**Figure 7:**
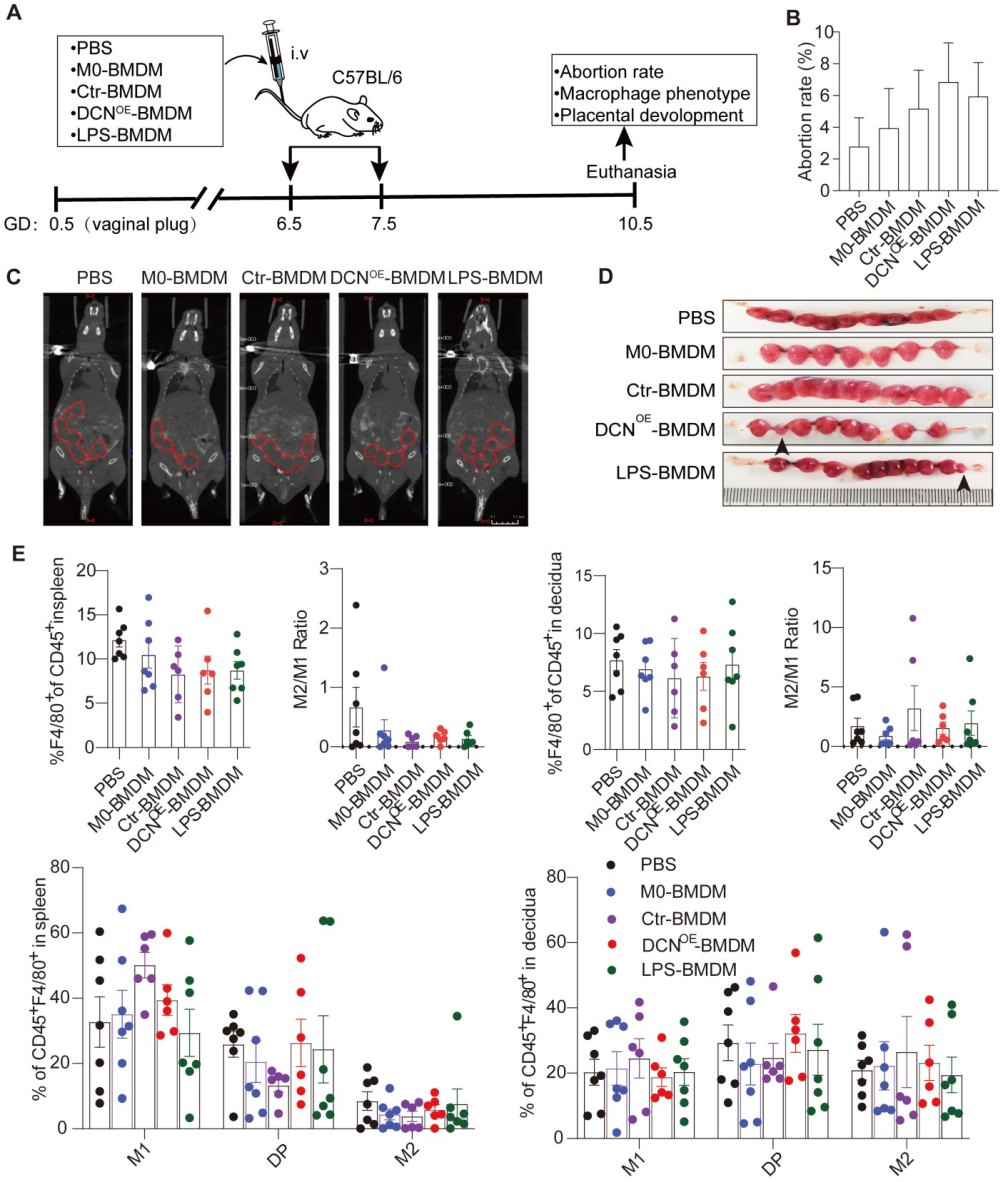
** Adoptive transfer of DCN-induced macrophages results in embryo absorption. (A)** C57BL/6 dams were randomly divided into five groups (phosphate-buffered saline [PBS], M0-BMDM, Ctr-BMDM, DCN^OE^-BMDM, and LPS-BMDM groups) and received the indicated BMDMs (2 ×10^6^ in 200 µL PBS) at GD6.5 and GD7.5. Dams in the PBS group were given 200 µL PBS at the indicated time. The embryo abortion rate, macrophage phenotype in the spleen and uterus, and placental development were evaluated on GD 10.5. **(B-D)** Embryo absorption rate in different groups was determined on GD10.5. Positron emission tomography (PET)-computed tomography (CT) imaging was used to identify the successful pregnancy in each group (C). Embryo absorption can be identified by hemorrhagic spots or small embryo size (D, black arrows, left). **(E)** Flow cytometry analysis of the proportion of total macrophages and macrophage subsets in the spleen and uterus.

To specifically address whether DCN-induced M1-like macrophages are responsible for pregnancy loss, we evaluated embryo absorption on GD10.5 after adoptive transfer of BMDMs with different treatments. BMDMs were treated with complete medium containing 20% L929 cell lines (M0-BMDMs), supernatant from ctr-293T cell lines (Ctr-BMDMs), or DCN^OE^-293T cell lines (DCN^OE^-BMDMs) *in vitro* and adoptively transferred to wild-type C57BL/6 dams on GD6.5 and GD7.5 (**Figure 7A**). We observed that adoptive transfer of DCN^OE^-BMDMs increased the embryo abortion rate (**Figure 7B-D**), accompanied by a slight reduction in the percentage of F4/80 in CD45^+^ leukocytes and the M2/M1 ratio in the spleen compared to that in the PBS group (**Figure 7E**). No significant changes were observed in the proportion of CD45^+^F4/80^+^ decidual macrophages, decidual M2/M1 ratio, or percentage of macrophage subsets (M1-like, M2-like, and double-positive macrophages) (**Figure 7E**).

**Figure 8:**
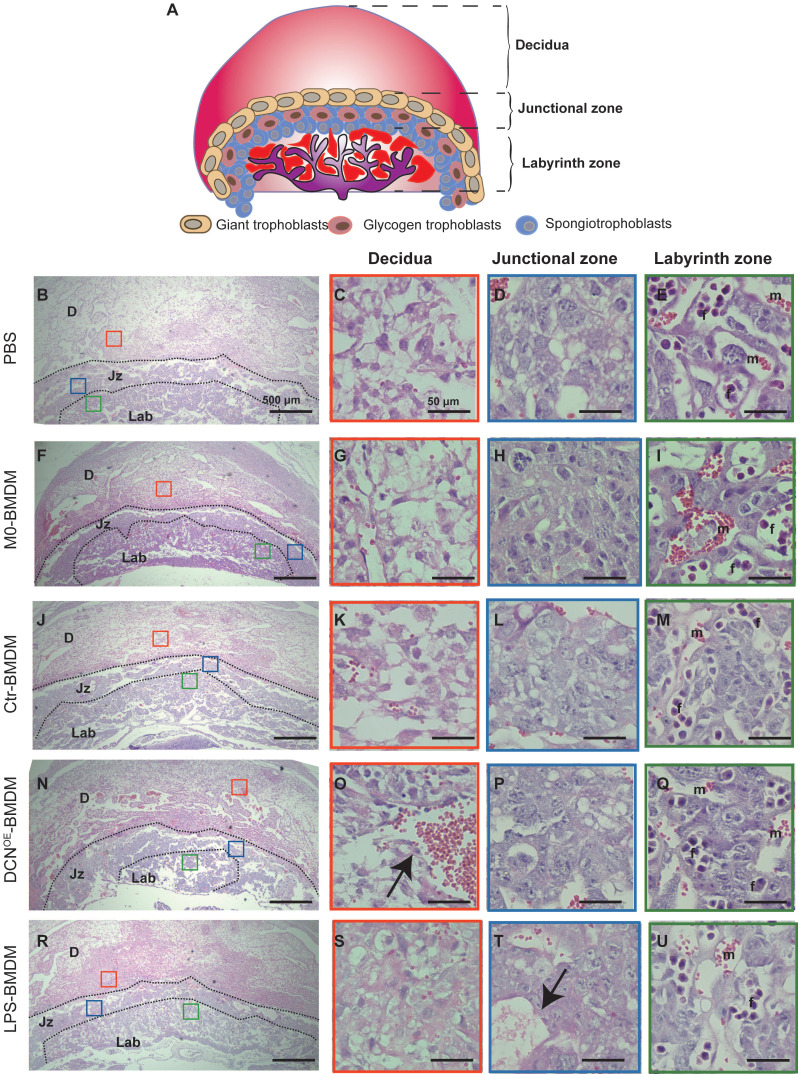
** Adoptive transfer of DCN-induced macrophages results in aberrant placental development. (A)** Schematic representation of mouse placental development on GD10.5, consisting of three compartments: maternal decidua, junctional zone, and labyrinth zone. **(B-U)** Hematoxylin and eosin (H&E)-stained cross sections of placentas from different groups on GD10.5. Red, blue, and green boxes (**B, F, J, N, and R**) indicate regions of higher magnification in panels to the right depicting the (**C, G, K, O, and S**) decidua, (**D, H, L, P, and T**) junctional zone, and (**E, I, M, Q, and U**) labyrinth zone, respectively. D: decidua; Jz: junctional zone; Lab: labyrinth zone; m: maternal blood sinusoid; f: fetal capillary. Scale bar: 500 or 50 µm.

Fetal vascularization is essential for normal fetal development. To assess whether adoptive transfer of BMDMs altered fetal vascularization, H&E staining and immunohistochemistry of laminin expression were conducted in placental sections. The placenta from different groups exhibited normal structures in the three compartments (maternal decidua, junctional zone, and labyrinth zone) (**Figure 8**). However, the area of the labyrinth zone was reduced in the DCN^OE^-BMDM group compared with that in the PBS placenta (**Figure 8N**). Increased maternal blood vessels in the decidua and decreased fetal blood vessels in the labyrinth zone were also observed in the placenta of the DCN^OE^-BMDM group than in the PBS, M0-BMDM, and Ctr-BMDM groups (**Figure 8C-Q**). The maternal decidua appeared to be more compact, with more vacuolation between the spongiotrophoblasts in the placenta of the LPS-BMDM group on GD10.5 (**Figure 8S-T**) compared with the PBS, M0-BMDM, and Ctr-BMDM groups (**Figure 8C-Q**).

**Figure 9:**
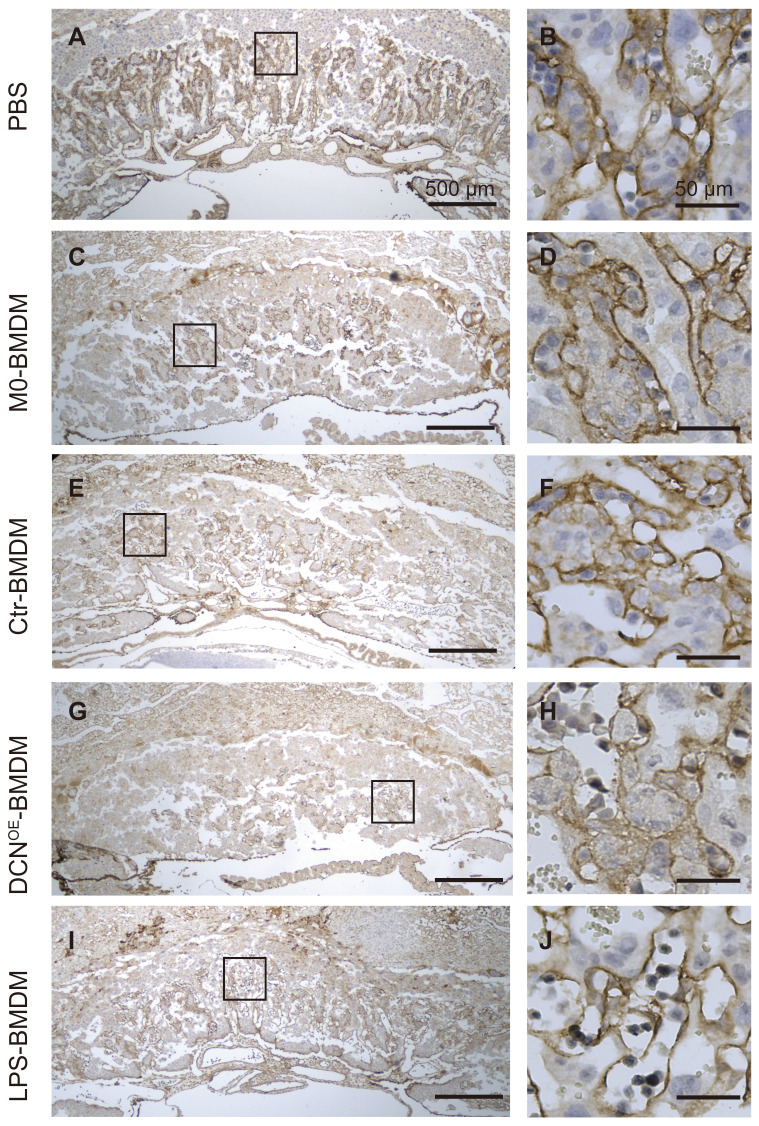
** Adoptive transfer of DCN-induced macrophages results in impaired fetal vascularization.** Immunohistochemistry of laminin was conducted to assess the fetal vascularization. Boxes in left images indicate the regions of higher magnification in the right panels in the labyrinth zone. Scale bar: 500 or 50 µm.

In the labyrinth zone of the DCN^OE^-BMDM group on GD10.5, laminin (a marker of the fetal endothelium and its associated basement membrane) immunostaining revealed defective fetal vascularization of the labyrinth. The walls of fetal capillaries were irregularly shaped, with less fetal vessel lumen, in contrast to their smooth and regular appearance in the other groups (**Figure 9**). These findings showed that DCN-induced M1-like macrophages were responsible for adverse pregnancy loss, which may be caused by impaired fetal vascularization.

## Discussion

Aberrant DCN levels are related to various pregnancy complications, including preeclampsia, fetal growth restriction, and preterm premature rupture of membranes 32. In this study, we demonstrated that high levels of DSC-derived DCN were detected in the decidua of women with RPL. Consistent with previous reports 33, 34, we found abnormal polarization of decidual macrophages in women with RPL, characterized by an increased percentage of M1-like decidual macrophages and decreased M2-like macrophages. Pearson's correlation analysis showed a positive correlation between decidual DCN levels and the proportion of M1-like decidual macrophages. An *in vitro study* demonstrated that exogenous DCN promotes macrophage polarization to M1-like macrophages. Our findings revealed that changes in mitochondrial metabolism were responsible for DCN-induced macrophage polarization, including enhanced glycolysis, increased mitochondrial fission, MMP, and cellular ROS levels, and activation of the MyD88-NF-κB signaling pathway. Moreover, the adoptive transfer of DCN-treated BMDMs led to an increased embryo abortion rate and impaired fetal vascularization. Thus, increased DCN levels may serve as one of the most important factors for aberrant M1-like macrophage polarization, resulting in the occurrence of RPL.

Previous studies have shown that DCN overproduction by decidual cells is associated with preeclampsia by reducing EVT migratory and invasive capacity, which contributes to insufficient conversion of maternal spiral arteries 35-37. Aberrantly high plasma DCN levels are predictive biomarkers of preeclampsia before clinical onset during the second trimester 14. The deficiency of trophoblast invasion observed in the decidua of women with RPL 38 may result from the high DCN content in the decidua. Moreover, prompt elimination of senescent DSCs by activated NK cells is important for decidualization, which controls embryo implantation. However, defects in this process predispose patients to RPL 39. An increased proportion of senescent DSCs leads to prominent secretion of various bioactive molecules, including extracellular matrix remodeling proteins, via a senescence-associated secretory phenotype 10, which may be responsible for DCN overexpression in the decidua of women with RPL. Other factors, such as the hyperactivation of matrix metalloproteinases (2, 3, and 7) 40, increased chemokine (CXCL13, CXCL16, and CCL5) levels 41, and Kang Ai 1 expression 42, could be involved in regulating DCN content in the decidua.

Our findings revealed that decidual DCN is mainly derived from DSCs, and its content presents dynamic changes with gestational weeks. Previous studies have revealed that DCN is produced by a variety of stromal cells in tissues, including decidua, fetal mesenchymal cells, fetal membrane, uterine cervix, and myometrium 32. However, single-cell RNA sequencing data suggest that DSCs contain the highest DCN mRNA content at the maternal-fetal interface during pregnancy 43. Consistent with our data, DCN production was enhanced in human endometrial stromal cells by stimulation with estrogen, progesterone and cAMP 15. DCN-depleted human endometrial stromal cells failed to mature fully in response to decidualizing stimuli, as demonstrated by fibroblastoid morphology, reduction in cell ploidy, and reduced ability to produce IGFBP1 and PRL 15, suggesting an autocrine role of DCN in decidualization. Additionally, it has been reported that the composition of the GAG chain and DCN content changes with gestational age in rodents 44, 45. A decrease in the proportion of CS to DS was detected in the rat placenta during late pregnancy, which might affect the binding affinity between DCN and collagen, thereby affecting the flexibility of the placenta 44. Similar to our data, Halari et al. 15 revealed that the levels of decidua-derived DCN mRNA significantly increased at eight weeks and remained stable until 11 weeks of gestation during early pregnancy, which may be attributed to estrogen, progesterone, and cAMP 42, 46. In addition, the metastasis suppressor CD82 expressed by decidual cells positively promotes DCN production 42. Except for the autocrine role of DCN in decidual cell maturation, our work, for the first time, revealed that changes in DCN levels are closely related to M1-like macrophage polarization during early pregnancy.

Similar to the results reported in previous studies 33, 34, our data revealed that the proportion of M1-like decidual macrophages was higher in women with RPL than in women with NP. More importantly, the decidual M2/M1 ratio fluctuates with gestational age during early pregnancy. Dynamic changes in macrophages are also observed in mice, as evidenced by a gradual reduction in the ratio of M1 to M2 macrophages during implantation 47. During early human pregnancy, the embryo develops under low oxygen conditions because of the clogged maternal arteries for the first 10-12 weeks of pregnancy and is supported by nutrients from uterine gland secretion, known as histotrophic nutrition. Subsequently, the trophoblast intravascular plugs dislodge after 10-12 weeks of pregnancy, allowing maternal blood to perfuse the intervillous space, increase oxygenation, and support the development of the embryo and placenta 1. A dramatic increase in oxygen levels during the establishment of placental circulation results in increased ROS production and oxidative stress 48. Increased ROS levels are involved in regulating the polarization and function of macrophages through various mechanisms, including promoting antibacterial responses and phagocytosis via H_2_O_2_ 49, 50, increasing proinflammatory cytokine production and iNOS protein expression through the MAPK and NF-κB signaling pathways, and enhancing LPS-induced anaerobic glycolysis and IL-1β secretion via stabilization of HIF-1α 31, 51. Thus, the specific change in oxygen levels, together with DCN, may collectively lead to a significant decrease in the M2/M1 ratio at 10 weeks of pregnancy.

Here, we also observed that exogenous DCN polarized macrophages toward M1-like macrophages, accompanied by proinflammatory cytokine overexpression, which is consistent with previous reports 19, 20. Mechanistically, the proinflammatory effect of DCN could be mediated by its interaction with TLR2/TLR4 or by the abolishment of TGF-β action. Activation of the MyD88-NF-κB signaling pathway upon DCN treatment in our study also supports the role of the interaction between DCN and TLR in M1-like macrophage polarization. Previous studies indicated that activated TLR2 and TLR4 at the maternal-fetal interface may lead to RPL by perturbing the Th1/Th2 immune response balance 52. Significantly higher frequencies of homozygous (2/2) TLR-2 rs1898830 genotype carriers have been observed in RPL cases than in control women 52. In addition, other studies have provided evidence of its proinflammatory role. Indeed, DCN plays an essential role in asthma, as DCN-deficient mice show reduced lung inflammation and increased expression of regulatory T cells and IL-10 mRNA 53. Based on the antitumor 54 and proinflammatory properties of DCN 20, oncolytic adenoviruses expressing DCN effectively inhibited tumor growth and metastasis in murine models of breast cancer 55 and renal cell carcinoma 56 by increasing Th1 cytokine expression and decreasing Th2 cytokines. Moreover, the combination of an oncolytic adenovirus carrying DCN and chimeric antigen receptor T cells targeting carbonic anhydrase IX (CAIX-CAR-T) exhibited a significantly reduced tumor burden, enhanced interferon (IFN)-γ secretion, and prolonged mouse survival compared to the CAIX-CAR-T treatment control 57. However, in a rat model of hyperoxic lung damage, DCN-derived mesenchymal stem cells promoted IL-10 secretion, increased CD163 expression, and inhibited IL-8 and IL-6 expression via CD44 expressed on macrophages, leading to the reconstruction of alveoli in damaged lung tissues 58. The paradoxical role of DCN may be related to the receptor and/or local microenvironment.

DCN-mediated M1-like macrophage polarization is related to alterations in mitochondrial metabolism, characterized by enhanced glycolysis, increased MMP and cellular ROS levels, and the promotion of mitochondrial fragmentation. The roles of DCN in mitophagy and autophagy have been determined in triple-negative breast carcinoma 59, endothelial 60, and glioma cells 61. The soluble DCN protein core suppresses OXPHOS with a concurrent significant loss of MMP and evokes mitochondrial fragmentation and mitophagy via the DCN/Met/PGC-1α/mitostatin axis in breast carcinoma cells 59. Moreover, the DCN protein core also induces autophagy and tilts the mitochondrial dynamic balance toward fission, in addition to decreasing MMP in a VEGFR2-dependent manner in endothelial cells 60. Indeed, we found a marked increase in the mitochondrial fission-associated protein phosphorylated DRP1 in response to DCN treatment, although the mitochondrial fusion-associated protein levels showed no changes. Moreover, DCN is specifically induced by nutrient deprivation and autophagic stimuli 62. Mice lacking DCN display impaired cardiac autophagy 62 and aberrant cardiac glucose utilization 63, following fasting compared to wild-type mice, protecting against reduced ejection fraction. It is worth noting that these aforementioned studies almost always chose a DCN protein core without GAG chains, as GAG chains are dispensable for its autophagic activity 11. However, previous studies have demonstrated that only intact DCN, not the protein core or the GAG chain of DCN, could trigger proinflammatory cytokine secretion in macrophages 20. Collectively, the different effects and signaling pathways mediated by DCN may be related to different receptors, cell subsets, and intact DCN.

Although the molecular mechanisms remain unclear, shifts in mitochondrial metabolism likely dictate the macrophage functions 23. M1 macrophages induced by LPS and IFN-γ are characterized by increased glycolysis and pentose phosphate pathway, accompanied by breaks in the tricarboxylic acid (TCA) cycle 64. These metabolic properties of M1 macrophages promote the production of proinflammatory IL-1β 30, 65, 66 and TNF-α 67 and enhance bacterial killing via mitochondrial ROS 30, 50. Consistently, increased mitochondrial oxidation of succinate evoked by the disruption of TCA cycle and elevation of MMP jointly drive mitochondrial ROS production 30, which finally promote LPS-mediated M1-like macrophage polarization. In addition, LPS-induced iNOS generates a large amount of nitric oxide, which prevents the repolarization of proinflammatory M1 to the anti-inflammatory M2 phenotype by dampening mitochondrial OXPHOS 25, 68. Mitochondrial structures are highly dynamic and correlated with the function of immune cells 23, 69. LPS triggers TLR4- and MyD88-dependent mitochondrial fission and inflammatory response by regulating the phosphorylation of DRP1, accompanied by enhanced glycolysis 70. Silencing DRP1 in macrophages can decrease TNF-α production via post-transcriptional modification during sterile inflammation or bacterial infection 71. Although the underlying mechanism needs to be further investigated, DCN may regulate macrophage polarization by altering the mitochondrial metabolism.

This study has some limitations. Although the mouse model confirmed the *in-vivo* effects of DCN-induced M1-like macrophages on embryo absorption and placental development, the detailed mechanisms were not elucidated in the mouse models. Moreover, the sample size in the RPL group was relatively small. Therefore, further investigations with a larger sample size are necessary to determine the underlying mechanism and validate our findings.

## Conclusions

**Figure 10:**
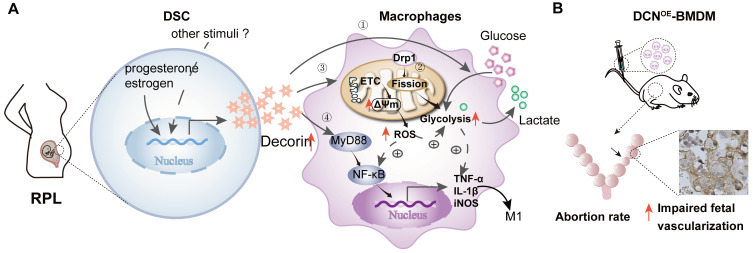
** DCN promotes decidual M1-like macrophage polarization via mitochondrial dysfunction resulting in the occurrence of RPL. (A)** Aberrant high levels of DCN, which is secreted by DSCs and induced by progesterone and estrogen, lead to the dysfunction of decidual macrophages in the decidua of women with RPL. Dysfunction of decidual macrophages is characterized by an increasing proportion of M1-like macrophages, accompanied by TNFα and IL-1β production. Notably, this polarization to M1-like macrophages is related to increasing mitochondrial membrane potential and glycolysis, promoting mitochondrial fission mediated by DRP1, enhancing ROS production, and activating the MyD88-NF-κB signaling pathway. **(B)** Transfer of DCN-treated BMDMs increases the mouse embryo absorption during early pregnancy, accompanied by impaired fetal vascularization.

In conclusion, our findings demonstrate for the first time that DSC-derived DCN is highly expressed in the decidua of women with RPL. Our *in-vitro* studies indicated that DCN treatment inhibited macrophage polarization to the M2-like phenotype and boosted the inflammatory response, which was related to enhanced anaerobic glycolysis, increased MMP and intracellular ROS levels, reduced mitochondrial mass, and activation of the MyD88-NF-κB signaling pathway. The *in-vivo* mouse model further confirmed the role of DCN-mediated M1-like macrophages in the occurrence of RPL, as evidenced by the increased embryo absorption and impaired fetal vascularization (**Figure 10**). Our findings provide novel insights into the factors mediating aberrant macrophage polarization, which may be used to develop new strategies for the diagnosis and advanced intervention of RPL.

## Supplementary Material

Supplementary figures and tables.

## Abbreviations

BMDMs: bone marrow-derived macrophages
CS: chondroitin sulfate
Drp1: dynamin-related protein 1
DS: dermatan sulfate
DSCs: decidual stromal cells
FAO: fatty acid oxidation
GAG: glycosaminoglycan
IGFBP-1: insulin-like growth factor binding protein 1
MFN: mitofusin
MMP: mitochondrial membrane potential
NP: normal pregnancy
OPA1: optric atrophy 1
OXPHOS: mitochondrial oxidative phosphorylation
RPL: recurrent pregnancy loss
PRL: prolactin
TCA: tricarboxylic acid
