# Supplementary material for: Decorin promotes decidual M1-like macrophage polarization via mitochondrial dysfunction resulting in recurrent pregnancy loss
Source: Theranostics. 2022 Oct 17;12(17):7216–36. doi: 10.7150/thno.78467 (PMC9691373; doi:10.7150/thno.78467)
Supplement: Supplementary file 1 — Supplementary figures and tables. [file thnov12p7216s1.pdf]

Supplemental Table 1 The specific antibodies used for flow cytometry

Antibodies	Identifier	Source
Human Tristan FcX™	422302	Biolegend
Mouse -anti human CD45 monoclonal antibody, APC	304012	Biolegend
Mouse -anti human CD14 monoclonal antibody, FITC	11-0149-42	Thermo
Mouse -anti human CD206 (MMR) monoclonal antibody, PE- Cyanine7	25-2069-42	Thermo
Mouse -anti human CD86 (B7-2) monoclonal antibody, PE	12-0869-42	Thermo
Rat anti-mouse CD16/CD32 monoclonal antibody	553142	BD biosciences
Rat anti-mouse CD45 monoclonal antibody, APC	103112	Biolegend
Rat anti-mouse F4/80 monoclonal antibody, FITC	123108	Biolegend
Rat anti-mouse CD86 monoclonal antibody, PE	105008	Biolegend
Rat anti-mouse CD206 (MMR) monoclonal antibody, PE/Cy7	141720	Biolegend

Supplemental Table 2 The specific antibodies used for western blotting, immunohistochemistry and immunofluorescence staining

Antibodies	Identifier	Source
Rabbit polyclonal anti-decorin	14667-1-AP	Proteintech
Gambit monoclonal anti-CK7	ab181598	Abcam
Mouse monoclonal anti-vimentin	60330-1-Ig	Proteintech
mouse monoclonal anti-CD68	Sc-20060	Santa
Rabbit polyclonal anti-CD163	ab182422	Abcam
Rabbit polyclonal anti-CD206	ab64693	Abcam
Rabbit polyclonal anti-INOS	ab3523	Abcam
Rabbit polyclonal anti-laminin	ab11575	Abcam
Goat polyclonal anti-CCR7	NB100-712	Novus Biologicals
Goat polyclonal anti-ARG1	ab92274	Abcam
Rabbit polyclonal anti-DRP1	12957-1-AP	Proteintech
Rabbit polyclonal anti-phospho-DRP1(S616)	PA5-64821	ThermoFisher
Rabbit polyclonal anti-OPA1	27733-1-AP	Proteintech
Rabbit polyclonal anti-MFN1	13798-1-AP	Proteintech
Rabbit polyclonal anti-MFN2	12186-1-AP	Proteintech
Rabbit polyclonal anti-MyD88	AF5195	Affinity
Rabbit polyclonal anti-phosphor-NF-κB p65 (Ser536)	AF2006	Affinity
Rabbit polyclonal anti-NF-κB p65	10745-1-AP	Proteintech
Mouse monoclonal anti-β-actin	66009-1-Ig	Proteintech
Goat anti-mouse IgG (488)	A23210	Babine
Goat ani-rabbit IgG (594)	A23420	Abbkine
Donkey anti-goat IgG (488)	ab150129	Abcam
Donkey anti-mouse IgG (555)	ab150106	Abcam
Goat anti-rabbit HRP	SA00001-1	Proteintech
Rabbit anti-goat HRP	SA00001-4	Proteintech

Supplemental Table 3 The primer sequences used in the study

Primer	Forward	Reverse	Product（bp）
Human-*DCN*	GACAACAACAAGC TTACCAGAGTA	TGAAAAGACTCAC ACCCGAATAAG	163
Human-*PRL*	GCTGTAGAGATTG AGGAGCA	CATTTTCTTTGGTT TCAGGA	88
Human-*IGFBP1*	TCACAGCAGACAG TGTGAGAC	CCCAGGGATCCTCT TCCCAT	88
Human-*β-ACTIN*	CATGTACGTTGCTA T CCAGGC	CTCCTTAATGTCAC GCAC GAT	250
Mouse-*Inos*	GTTCTCAGCCCAA CAATACAAGA	GTGGACGGGTCGA TGTCAC	127
Mouse-*Arg1*	CCACAGTCTGGCA GTTGGAAG	GGTTGTCAGGGGA GTGTTGATG	106
Mouse-*Il-10*	GAGAAGCATGGCC CAGAAATC	CGCATCCTGAGGG TCTTCA	71
Mouse-*Tgf-β1*	CAACAATTCCTGGC GTTACCTTGG	GAAAGCCCTGTATT CCGTCTCCTT	128
Mouse-*Il-1β*	TGGACCTTCCAGG ATGAGGACA	GTTCATCTCGGAGC CTGTAGTG	148
Mouse-*Tnf-α*	CCCTCACACTCAG ATCATCTTCT	GCTACGACGTGGG CTACAG	61
Mouse-*β-actin*	GTGACGTTGACATC CGTAAAGA	GCCGGACTCATCG TACTCC	245

Supplemental Table 4 Characteristics of women from the NP and RPL groups for flow cytometry

	**NP groups** （**n = 30**）	**RPL groups** （**n = 21**）	***P* value**
**age**（**y**）	29.96 ± 3.58	31.76 ± 4.31	> 0.05
**BMI**（**kg/m^2**）	20.53 ± 1.26	21.56 ± 2.39	> 0.05
**Gestational day**	66.83 ± 6.75	71.32 ± 11.52	> 0.05
**Gravidity**	2.00 (2.00)	2.00 (2.00)	> 0.05
**Spontaneous abortion times**	/	2.00 (1.75)	/
**twice**	/	57.14%	/
**Third times**	/	19.05%	/
**Four times**	/	14.29%	/
**Five times**	/	9.52%	/

Note: Data are analyzed by Student's *t* test in mean±SEM or by the Mann–Whitney U test in median and IQR. NP, normal pregnancy; RPL, recurrent pregnancy loss. *P < 0.05. /: not applicable.

Supplemental table 5 Characteristics of 126 women from normal early pregnancy

Gestational weeks（w）	5 w (n = 8)	6 w (n = 35)	7 w (n = 31)	8 w (n = 28)	9 w (n = 12)	10 w (n = 9)	11 w (n = 3)	*P* value
age（y）	30.88 ± 6.13	29.09 ± 5.96	26.32 ± 4.14	27.92 ± 6.31	27.55 ± 4.99	28.33 ± 5.16	32.00 ± 3.27	> 0.05
Gravidity	3.00 (1.00)	3.00 (2.00)	2.00 (2.00)	2.00 (1.00)	2.00 (1.75)	2.00 (2.00)	2.00 (1.00)	> 0.05
Parity	1.00 (2.00)	1.00 (0.00)	0 (1)	1.00 (1.00)	1.00 (1.00)	0.00 (1.00)	1.00 (0.50)	> 0.05
Abortion for elective termination	0.00	1.00 (2.00)	0 (1.00)	0.00 (1.00)	0 (0.75)	0.00 (1.00)	0.00 (0.50)	> 0.05

Note: Data are analyzed by Student's *t* test in mean±SEM or by the Mann–Whitney U test in median and IQR. **P* < 0.05.

Supplemental table 6 The proportion of total decidua macrophages and decidua macrophage subsets with increasing gestational weeks during normal early pregnancy

Gestational weeks (w)	5 w	6 w	7 w	8 w	9 w	10 w	11 w
$CD45^+CD14^+$ (%)[a]	13.22 ± 7.27	23.40 ± 10.84	21.10 ± 9.13	22.66 ± 10.33	25.36 ± 11.43	25.12 ± 11.29	29.82 ± 10.7
M1(%)	14.97 ± 11.69	17.25 ± 7.56	13.75 ± 10.13	13.16 ± 11.15	6.57 ± 6.47[b]	17.12 ± 13.0	5.76 ± 4.30
M2(%)	45.30 ± 13.11	29.93 ± 15.82	28.70 ± 17.20	34.88 ± 21.45	44.55 ± 26.50	24.88 ± 14.08	42.35 ± 20.16
Double positive (%)	21.54 ± 3.43	36.79 ± 20.57	41.24 ± 23.83	38.28 ± 22.89	32.50 ± 29.52	31.10 ± 11.71	39.52 ± 23.69
M2/M1 ratio	5.33 ± 3.97	2.20 ± 1.66	5.02 ± 7.99	27.32 ± 80.02	65.48 ± 116.28	6.53 ± 10.50	19.97 ± 20.25

Note: Data are analyzed by one-way ANOVA in mean±SEM. [a] indicates the percentage of $CD45^{+}CD14^{+}$ in total leukocytes. [b] Indicates statistically significant differences at 9 w *vs*. 6 w.

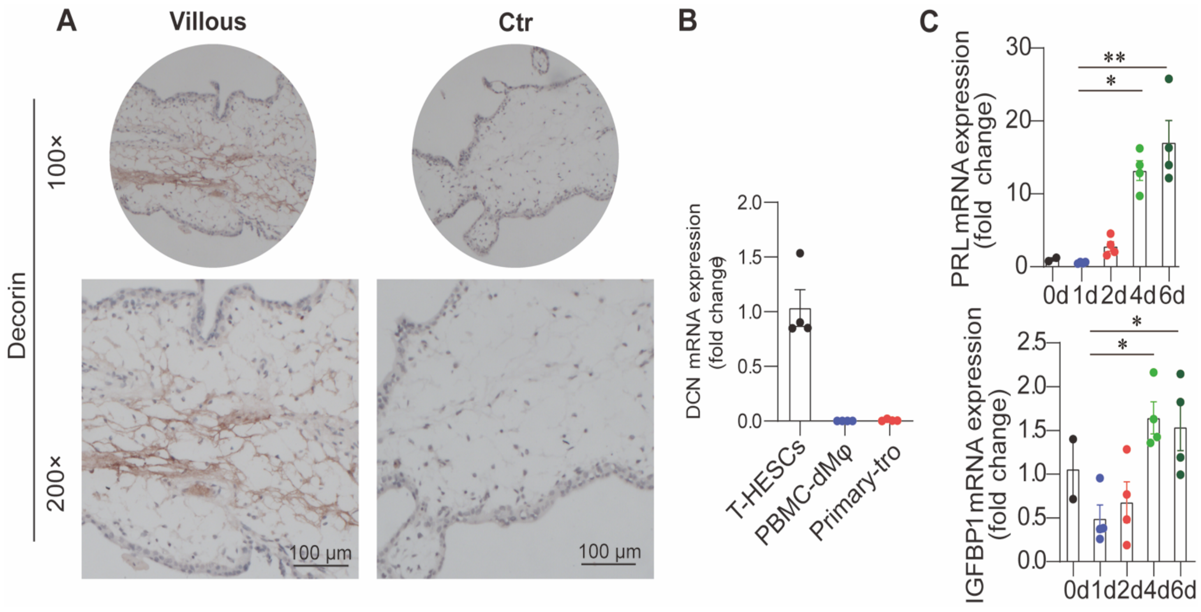

Figure S1 Decorin is mainly secreted by DSCs (A) The localization of decorin in villous of normal early pregnancy and RPL group by immunochemistry. Scale bar: 100 am. (B) RT–qPCR analysis of *DCN* mRNA in T-HESCs (day 6 after decidualization *in vitro*), PBMC-dM cells and primary trophoblasts (n = 4). PBMC-dM: peripheral blood monocyte cell-derived macrophages. PBMCs were sorted by MACS using CD14 microbeads and treated with 15 ng/ml M-CSF and 20 ng/ml IL-10 for 7 days *in vitro*. Primary trophoblasts were isolated from villous of normal early pregnancy. (C) PRL mRNA and IGFBP1 mRNA levels in T-HESCs were determined at the indicated time points (day 0 (0 d), 1 d, 2 d, 4 d and 6 d) after decidualization. (B, C) Data represent the mean ± SEM and were analyzed by one-way ANOVA. *$P < 0.05$, **$P < 0.01$.

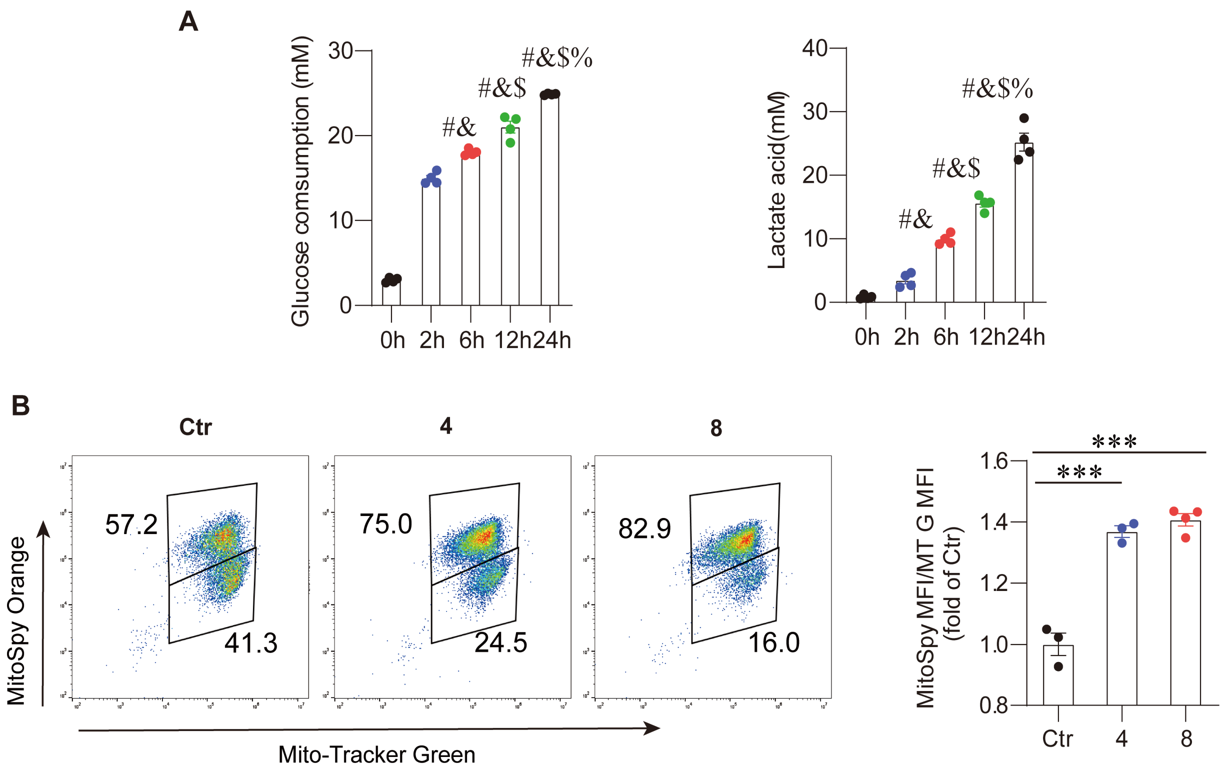

Figure S2 Dysfunctional mitochondrial metabolism determines the inflammatory phenotype of decorin-treated macrophages (A) Analysis of glucose consumption and lactate production in the supernatant of decorin-treated Raw264.7 cells at the indicated time points. (n =4 for each group). #, &, $, % indicate statistically significant differences *vs.* 0 h, 2 h, 6 h and 12 h, respectively. (B) MitoSpy- and MTG-stained cells were tested by flow cytometry, and MMP was identified as the MitoSpy MFI/MTG MFI ratio. Data represent the mean ± SEM and were analyzed by one-way ANOVA. *$P < 0.05$, ***$P < 0.001$.
